# Genetic Variations in the Folate Regulatory Enzyme ALDH1L1: Impact on Cell Metabolism, Proliferation, and Motility

**DOI:** 10.3390/cells15212026

**Published:** 2026-11-06

**Authors:** Halle M. Meyers, Amira A. Abdellatef, Natalia I. Krupenko, Baba Mass, Venkata Saroja Voruganti, Susan Sumner, Blake R. Rushing, Sergey A. Krupenko

**Affiliations:** 1 Nutrition Research Institute, UNC–Chapel Hill, Kannapolis, NC 28081, USA; halle_fogle@unc.edu (H.M.M.); amira_abdellatef@unc.edu (A.A.A.); natalia_krupenko@unc.edu (N.I.K.); bmass@live.unc.edu (B.M.); saroja@unc.edu (V.S.V.); susan_sumner@unc.edu (S.S.); blake_rushing@unc.edu (B.R.R.); 2 Department of Nutrition, UNC–Chapel Hill, Chapel Hill, NC 27599, USA

**Keywords:** ALDH1L1, exonic SNPs, untargeted metabolomics, tumor suppression, cancer metabolism

## Abstract

**Highlights:**

**What are the main findings?**

- ALDH1L1 haplotype variants differ in their ability to regulate metabolism.
- ALDH1L1 variants have diminished effects on proliferation and motility.

**What are the implications of the main findings?**

- ALDH1L1 haplotypes produce distinct metabolic and cellular phenotypes in cancer cell models.
- Metabolic responses to ALDH1L1 variants are influenced by cellular genetic background.

**Abstract:**

In humans, six common non-synonymous exonic single-nucleotide polymorphisms in *ALDH1L1* lead to ten enzyme haplotypes. Since *ALDH1L1* is a metabolic regulator and a candidate tumor suppressor, we studied how haplotype variants affect metabotypes, proliferation and motility. Using lentiviral delivery to A549 and PC3 cells, we have generated two cell panels, with each cell line in the panel carrying one of ten *ALDH1L1* haplotype variants. Untargeted metabolomics revealed strong differences in metabotypes between cells carrying *ALDH1L1* variants. Certain *ALDH1L1* variants had decreased ability to regulate one-carbon metabolism and downstream pathways, including amino acid and glutathione metabolism, nucleotide biosynthesis, and the TCA cycle. The direct comparison of A549 and PC3 cells using an untargeted metabolomic dataset also showed that the two cell lines have vastly different metabotypes. In agreement with this finding, the analysis of A549 and PC3 *ALDH1L1* haplotype panels suggested that the *ALDH1L1*-linked metabolic regulation is strongly influenced by specific cellular metabotypes and genetic background. *ALDH1L1* variants also exhibited reduced inhibition of cellular proliferation and motility compared to the most common haplotype, which supports further investigation of their potential relevance to individual risk of metabolic disorders.

## 1. Introduction

Folate (vitamin B9) is a group of coenzymes functioning as carriers of one-carbon (1C) groups in multiple biochemical pathways [1]. Folate-dependent reactions are of critical importance for amino acid metabolism, nucleotide biosynthesis, and methylation processes [2]. Additional roles for folate coenzymes in energy production and mitochondrial protein biosynthesis have also been underscored [3,4,5]. Because of such a broad function in the cell, deficiencies in folate metabolism can produce various adverse effects and increase the risk of several diseases, most notably neural tube defects and cancer (Reviewed in [2,6,7,8,9]). Key factors supporting folate metabolism are the availability of folate coenzymes for the cell and the activity of enzymes of folate-dependent pathways. Folate metabolism involves more than 20 enzymes, whose roles were established by extensive research (reviewed in [10,11]). While aberrant expression of folate enzymes has been linked to human disease, most commonly cancer [12,13], significant individual variabilities in expression or activities of several such enzymes have also been reported [14,15,16,17]. One of the mechanisms accounting for individual variabilities in folate cycle enzymes is genetic variations in corresponding genes, most commonly single nucleotide polymorphisms (SNPs) [18,19,20]. The best studied example is the C677T polymorphism in *MTHFR*, which causes a non-synonymous amino acid substitution, creating an enzyme variant with reduced activity [21]. Meta-analyses indicated that there is an inverse association between the *MTHFR C677T* genotype and the risk of colorectal cancer [22,23,24]. SNPs in other genes of 1C metabolism, including *DHFR* [25], *TS* [26] and *MTR* [27], can affect the expression/function of corresponding proteins and play a role in susceptibility to tumorigenesis, cancer progression or response to chemotherapies. Overall, genetic polymorphisms in folate-metabolizing enzymes are linked to genome integrity and epigenetic regulation, thereby contributing to cancer susceptibility [19,28]. Importantly, interactions between these polymorphisms and diet may synergistically promote malignant transformation [19].

Non-synonymous SNPs are also common in the *ALDH1L1* gene, which encodes cytosolic 10-formyltetrahydrofolate dehydrogenase [18]. It is an abundant enzyme of folate metabolism with a regulatory function [29]. It catalyzes the NADP^+^-dependent conversion of 10-formyltetrahydrofolate to tetrahydrofolate, in which formyl, the folate-bound carbon group, is oxidized to CO_2_. This reaction is catabolic in nature, and it irreversibly removes 1C from the reduced folate pool [30]. In the folate cycle, the ALDH1L1 reaction regulates de novo purine biosynthesis, serine to glycine conversion, and the flux of 1C groups towards methylation [31,32,33,34,35]. Because the enzyme prevents the overflow of 1C groups towards biosynthetic reactions, it is frequently downregulated or silenced in cancer (reviewed in [30]). This silencing is primarily driven by DNA methylation in the promoter region, first exon, and downstream intron [36,37,38]. In support of ALDH1L1’s role in the regulation of proliferation, the enzyme is also downregulated through proteasomal degradation during the S-phase of the cell cycle [39]. ALDH1L1 downregulation apparently removes the control from the folate-dependent biosynthetic processes, supporting limitless proliferation. Thus, ALDH1L1 has been considered as a candidate tumor suppressor based on its loss in malignant tumors [18,40,41].

Although the role of SNPs in ALDH1L’s function as a tumor suppressor has not been directly investigated, an epidemiological study linked a non-synonymous exonic ALDH1L1 SNP, rs1127717, to an increased risk of hepatocellular carcinoma in a Chinese population [42]. Another such SNP, rs2276724, was associated with the postoperative survival of patients with hepatitis B-related hepatocellular carcinoma [43]. Overall, there are six common non-synonymous exonic SNPs in the *ALDH1L1* gene (Figure 1A) [18]. Amino acid substitutions caused by these SNPs are spread across all three ALDH1L1 functional domains (Figure 1B). Several of these SNPs are often inherited together, leading to the ten most common *ALDH1L1* haplotypes (Figure 1A). Analysis of the NCI-60 panel as well as additional human cancer cell lines indicates the prevalence of exonic *ALDH1L1* SNPs (Supplementary Tables S1 and S2), raising the question of whether ALDH1L1 genetic variants are linked to cancer.

Typically, studies of genetic polymorphisms rely on the analysis of associations between disease/conditions and specific SNPs [44,45]. However, the six common non-synonymous *ALDH1L1* SNPs are frequently inherited in combination and give rise to ten major haplotypes (Figure 1A). A haplotype-based approach is therefore useful for evaluating the functional consequences of naturally occurring combinations of amino acid substitutions rather than considering each SNP independently. In the present study, we investigated the direct impact of *ALDH1L1* haplotype variants, associated with six common non-synonymous SNPs, on metabotypes of two cancer cell lines, A549 and PC3. Both cell lines lack ALDH1L1 expression due to extensive methylation of this gene promoter [36], which makes them suitable models for this approach. Thus, our study assessed the overall metabolic impact of specific *ALDH1L1* haplotype variants, compared variant-associated metabotypes, identified pathways under the control of ALDH1L1 in cancer cells, and evaluated cell proliferative responses in the presence of each specific enzyme variant.

## 2. Materials and Methods

### 2.1. Available Databases

Genetic variants in ALDH1L1 across the NCI-60 human cancer cell line panel were identified using the National Cancer Institute (NCI) CellMiner database (https://discover.nci.nih.gov/cellminer/; accessed on 21 July 2026). CellMiner is a publicly available resource that integrates genomic and pharmacological data from the NCI-60 cancer cell line panel. Expression levels of mRNA for selected folate-metabolizing enzymes in the A549 and PC3 cell lines were obtained from The Human Protein Atlas (https://www.proteinatlas.org/; accessed on 4 March 2026).

### 2.2. DNA Extraction and Genotyping Analysis of ALDH1L1 SNPs in Cancer Cells

Cells were cultured, and cell pellets were collected for genomic DNA extraction. Genomic DNA was isolated using the Forensic DNA Extraction Kit (BioChain Institue, Inc., Newark, CA, USA; #Z1322010) on the AnaPrep 12 automated DNA extraction platform according to the manufacturer’s instructions. DNA concentration and purity were assessed using a NanoDrop™ One spectrophotometer (Thermo Fisher Scientific, Waltham, MA, USA). Samples with A260/A280 ratios between 1.8 and 2.0 were considered suitable for downstream genotyping. Genomic DNA was diluted to a working concentration of 5 ng/µL and stored at −20 °C until analysis.

Pre-designed TaqMan™ SNP Genotyping Assays containing allele-specific probes labeled with VIC and FAM fluorescent dyes, along with primers flanking the target SNP loci, were used for genotyping. Reactions were prepared in a final volume of 10 µL in standard 96-well PCR plates, with each reaction containing 10 ng genomic DNA, 1× TaqPath™ ProAmp™ Master Mix, 1× SNP-specific TaqMan™ assay, and nuclease-free water. For each SNP assay, three positive control samples representing known genotypes were included to ensure assay performance and genotyping accuracy.

PCR amplification and allelic discrimination were performed using the QuantStudio™ 12K Flex Real-Time PCR System (Thermo Fisher Scientific, Waltham, MA, USA). Thermal cycling conditions consisted of a pre-read step at 60 °C for 30 s, an initial enzyme activation at 95 °C for 5 min, followed by 40 cycles of denaturation at 95 °C for 15 s and annealing/extension at 60 °C for 1 min. Genotypes were assigned using TaqMan™ Genotyper software version 1.4 based on relative VIC and FAM fluorescence intensities. Genotype clusters were visually inspected for quality and accuracy. Samples with ambiguous clustering or low signal intensity were excluded, and SNPs with call rates below 95% were removed from further analysis.

### 2.3. Cell Lines and Culture Conditions

A549, PC3, and HEK-293a cells (American Type Culture Collection [ATCC], Manassas, VA, USA) were obtained from the Tissue Culture Facility at the UNC Lineberger Comprehensive Cancer Center (Chapel Hill, NC, USA), which performs routine cell line authentication through commercial services (Labcorp, Burlington, NC, USA) using Short Tandem Repeat (STR) DNA profiling analysis.

Cells were cultured for no more than 10–20 sequential passages. A549 cells were cultured in F12K Nutrient Mixture Kaighn’s medium (Gibco, Thermo Fisher Scientific, Waltham, MA, USA; #21127-022). PC3 cells were cultured in RPMI 1640 medium (Gibco, #11875093). HEK-293a cells were cultured in DMEM medium (Gibco, Thermo Fisher Scientific, Waltham, MA, USA; #11885-084). All media were supplemented with 10% fetal bovine serum (Bio-Techne, Minneapolis, MN, USA; #S11150) and 1% antibiotic-antimycotic (Gibco, #15-240-062). All cells were maintained at 37 °C in an incubator with 5% CO_2_. All cell lines used in this study were mycoplasma-free, as tested using PCR Mycoplasma detection kit (Thermo Fisher Scientific, Waltham, MA, USA; #AAJ66117AMJ). All experiments were performed using three independent biological replicates unless otherwise indicated.

### 2.4. Plasmid Construction and Amplification

A custom lentiviral vector encoding human *ALDH1L1* cDNA was purchased from GeneCopoeia, Inc. (Rockville, MD, USA; #EX-Q0477-Lv102-B). The vector map and design have been described previously [46]. Briefly, the *ALDH1L1* open reading frame (ORF; NM_012190.3/XM_024453325.2) was cloned into the pReceiver-Lv201 vector downstream of the CMV promoter. The resulting construct co-expresses ALDH1L1 under the control of the CMV promoter and eGFP under the control of the SV40 promoter and contains a puromycin resistance cassette for selection.

### 2.5. Site-Directed Mutagenesis

Mutations in the lentiviral vector corresponding to human SNPs were introduced by site-directed mutagenesis using the QuickChange Kit (Agilent Technologies, Santa Clara, CA, USA; #200517) according to the manufacturer’s instructions. Sequences of oligonucleotides used for site-directed mutagenesis are listed in Table S3. Briefly, plasmids were PCR-amplified using an Analytik Jena Biometra TRIO 48 thermal cycler (Analytik Jena GmbH, Jena, Germany) with QuickChange Lightning Site-Directed Mutagenesis Kit reagents (Agilent Technologies, Santa Clara, CA, USA; #210519). The annealing temperatures were optimized according to primer-specific recommendations (Table S3). In the case of multiple nucleotide substitutions, site-directed mutations were performed in several steps. Following amplification, parental DNA was digested with 1 µL of DpnI restriction enzyme. The digested products were then transformed into XL10-Gold ultracompetent cells (Agilent, #200315) and plated on ampicillin-containing agar plates for overnight incubation. Individual bacterial colonies were selected and expanded in liquid culture, and plasmid DNA was purified using QIAprep Miniprep Kits (QIAGEN, Hilden, Germany; #27104) according to the manufacturer’s instructions. All plasmid constructs were verified by sequencing (Eurofins Genomics, Louisville, KY, USA).

### 2.6. Lentiviral Packaging and Transfection

Recombinant lentivirus was packaged in HEK-293a cells using the Lenti-Pac™ HIV Expression Packaging Kit (Genecopoeia, Inc., Rockville, MD, USA; LT002) according to the manufacturer’s protocol as we previously described [46]. Briefly, 2.5 μg of lentiviral vector was diluted in 200 μL of Opti-MEM^®^ I (Gibco, Thermo Fisher Scientific, Waltham, MA, USA; #A36351-01) and mixed with 5 μL (0.5 μg/μL) of Lenti-Pac HIV in a sterile polypropylene tube. In a separate tube, 15 μL of EndoFectin Lenti was diluted in 200 μL of Opti-MEM I. The diluted EndoFectin Lenti reagent was then added dropwise to the DNA solution while gently vortexing the tube. The mixture was incubated for 10–25 min at room temperature to allow the DNA-EndoFectin complex to form. HEK-293a packaging cells were transfected by adding the entire DNA-EndoFectin Lenti complex and were incubated overnight (8–14 h). Cells were placed in fresh medium supplemented with 1/500 volume of TiterBoost reagent and incubated for an additional 24 h. Pseudovirus-containing culture medium was collected in sterile capped tubes at 36 and 48 h post-transfection. The medium was centrifuged at 500× *g* for 10 min to remove cell debris, and the supernatant was filtered through 0.45 μm PES low protein-binding filters. Lentiviral titration was performed using the Lenti-Pac™ HIV qRT-PCR Titration Kit (GeneCopoeia, Rockville, MD, USA; LT005) according to the manufacturer’s instructions. A lentiviral MOI of 50 was used for transient transduction of A549 and PC3 cells in all experiments.

### 2.7. Cell Viability

We used the Cell Counting Kit-8 (CCK-8; Dojindo Molecular Technologies, Inc., Rockville, MD, USA; #CK04), a colorimetric assay measuring cellular metabolic activity, which correlates with the number of viable cells. Cells were plated in a 96-well plate at a density of 3 × 10^3^ cells/well and incubated overnight for attachment. CCK-8 reagent was added the next day according to the manufacturer’s instructions. Absorbance was measured at 450 nm using a Wallace 1420 Multilabel Counter (PerkinElmer, Waltham, MA, USA).

### 2.8. Colony Formation Assay

Cells seeded in a six-well plate (500–1000 cells per well) were cultured for 2 weeks. The medium was changed every 3–4 days. Then, the medium was aspirated, the cells were rinsed twice with PBS, and colonies were fixed with an ice-cold methanol-acetone mixture (1:1) and stained with 0.5% crystal violet (Sigma-Aldrich, St. Louis, MO, USA; #C0775-25G) for 1 h. The wells were rinsed carefully with clean water and then air-dried at room temperature. For each control or treatment, at least three wells were tested. Images of the entire plate were acquired using a camera. The colony number was counted using ImageJ, version 1.54g (National Institute of Health, Bethesda, MD, USA).

### 2.9. Wound Healing Assay

Cells were seeded at a density of 3 × 10^5^ cells/well in a 12-well plate and cultured for 24 h to form a monolayer (80–90% confluency). To minimize the contribution of cell proliferation to wound closure, cells were serum-starved for 24 h before scratching the monolayer. Scratches were generated using a sterile 100 μL pipette tip, and cells were then washed with PBS to remove detached cells and debris. Fresh medium (1 mL) was added to each well following washing. Images were captured immediately after scratching (0 h) and after 24 h and 48 h to assess the extent of wound healing. Wound closure was calculated using the following equation: wound closure (%) = [1 − (cell-free area at 48 h/cell-free area at 0 h)] × 100, with the cell-free area measured immediately after the scratch (0 h) and 48 h later. The wounded area was quantified manually using ImageJ software, version 1.54g (National Institute of Health, Bethesda, MD, USA).

### 2.10. Western Blot Analysis

Cell pellets were lysed using RIPA buffer containing a protease and phosphatase inhibitor cocktail (Thermo Fisher Scientific, Waltham, MA, USA; #89901). Total protein concentration was determined using a bicinchoninic acid (BCA) assay kit (Thermo Fisher Scientific, Waltham, MA, USA; #23225). A total of 45 μg of protein was separated by SDS-PAGE using an 8–16% precast Criterion gel (BioRad Laboratories, Hercules, CA, USA; #4561104) and transferred onto a polyvinylidene fluoride membrane (Thermo Fisher Scientific, Waltham, MA, USA; #88518). Membranes were blocked with 3% dry milk in TBST for at least 2 h at room temperature followed by overnight incubation of primary antibodies: In-house anti-ALDH1L1 antibody (1:8000); p-cofilin (Ser3) (Cell Signaling Technology, Danvers, MA, USA; #3311, 1:1000), which specifically recognizes phosphorylated cofilin at Ser3; Cofilin (D3F9) monoclonal antibody (Cell Signaling Technology, Danvers, MA, USA; #5175, 1:1000) was used to detect total cofilin at 4 °C). Secondary antibody incubation was performed using horseradish peroxidase (HRP)—conjugated anti—rabbit secondary antibody (Cytiva, Marlborough, MA, USA; #NA934V, 1:2000) or HRP-conjugated anti-mouse secondary antibody (Cytiva, Marlborough, MA, USA; #NA931V, 1:2000) for 1 h at room temperature. An HRP-conjugated anti-β-actin-specific antibody (clone AC-15; Abcam, Cambridge, UK; #AB49900, 1:20,000) was used as a loading control for all samples. For imaging, membranes were incubated with chemiluminescent substrate (Millipore, Burlington, MA, USA; #WBLUF0100) for approximately 2 min, and specific bands were visualized on an Odyssey FC Imaging System (LI-COR Biosciences, Lincoln, NE, USA). Protein bands were quantified using ImageJ Software, version 1.54g (National Institute of Health, Bethesda, MD, USA).

### 2.11. Metabolite Extraction for Untargeted Metabolomics

Metabolites were extracted from cells using previously described methods [46,47]. In brief, cells were grown in 10 cm dishes until they reached 70–80% confluency. Each haplotype expressed in A549 and PC3 cells was represented by five independent replicates. For harvesting, dishes were placed on ice, and cells were washed twice with 10 mL ice-cold PBS. To halt metabolic activity, 1 mL of ice-cold acetonitrile was added. After the addition of 750 μL ice-cold LC-MS-grade water, cells were dislodged by scraping, and cell suspensions were transferred to 15 mL conical tubes. These steps were repeated an additional time to harvest all remaining cells on the plate. Cells were centrifuged at 16,000× *g* for 10 min at 4 °C. The supernatant was then dried using a SpeedVac system and reconstituted in a 95:5 water:methanol solution according to each sample’s calculated protein concentration. A quality control study sample (QCSP) was generated by mixing 10 μL of each individual sample. LC-MS-grade water was used to prepare blank samples, which were processed using the same procedure as the study samples.

### 2.12. Analysis of Untargeted Metabolomics

Metabolomics data were obtained using a Vanquish UHPLC system coupled to an Orbitrap Exploris 240 Mass Spectrometer (Thermo Fisher Scientific, Waltham, MA, USA), following previously reported methods [34,48]. Blanks and QCSPs were interspersed among the randomized study samples at a rate of 10% during data acquisition. Data were preprocessed using Progenesis QI (Nonlinear Dynamics, Waters Corporation, Newcastle upon Tyne, UK) and filtered by removing peaks with a higher average abundance in the blanks compared with the QCSPs. Data were normalized to the total intensity of each sample. Identification and annotation of peaks to metabolites were performed previously described using an evidence-based ontology system that communicates the extent to which peaks match compounds based on retention time, exact mass, and MS/MS fragmentation patterns [34,48]. No filtering was performed based on missing values, and no log transformation was applied to peak intensities. Peak intensities of missing values were represented as zero values, and no further imputation was performed. All univariate and multivariate analyses were performed with zero values in the dataset. All multivariate models were performed using autoscaled data. In experiments for ALDH1L1 haplotype analysis, A549 and PC3 metabolomics samples were processed and acquired in separate runs on the same instrument using identical method parameters. No common pooled QC sample was injected across the two runs, and no formal batch-correction procedure was applied. Instead, statistical comparisons were performed independently within each cell line, and only summary-level results (e.g., significantly altered pathways) were compared between cell lines. The number of features differed between the A549 and PC3 runs (12,583 vs. 23,238) which may reflect differences in cell type, culture medium, and run-specific alignment and peak picking. Analyses were therefore restricted to OL1-PDA-annotated metabolites, and all statistical comparisons were performed within each cell line.

In a separate experiment, untargeted metabolomics was performed simultaneously for A549 and PC3 cells. Cell growth conditions and metabolite extraction were as described above. Metabolite extraction from A549 and PC3 cells was carried out in parallel, and the analysis of all samples was performed in a single run as described above.

### 2.13. Statistical Analysis

Statistical analyses were performed using Student’s *t*-test, one-way ANOVA, or two-way ANOVA, with *p* < 0.05 considered statistically significant. All analyses were conducted using GraphPad Prism (version 10.4.0). Untargeted metabolomics data were processed and analyzed using MetaboAnalyst 6.0 [49] and SIMCA 18.0 (Sartorius Stedim Data Analytics, Umeå, Sweden). Multivariate analyses, including principal component analysis (PCA) and orthogonal partial least squares-discriminant analysis (OPLS-DA), were used to evaluate group separation. OPLS-DA models were built in SIMCA 18.0 using a dummy Y matrix with one binary column per class. R^2^ and Q^2^ (calculated from a 7-fold cross validation) for all supervised multivariate models are provided in Supplementary Table S4. Pathway enrichment analysis was performed using the Pathway Analysis module in MetaboAnalyst 6.0. Input metabolites were selected based on matching to an in-house library by retention time and a *t*-test *p*-value < 0.05 between all groups. Correction for multiple testing was performed for all *p*-values using False Discovery Rate (FDR) correction, with FDR-adjusted *p* < 0.05 considered statistically significant.

## 3. Results

### 3.1. Expression of ALDH1L1 Haplotype Variants in A549 and PC3 Cells

A549 and PC3 cell lines lack the expression of endogenous ALDH1L1 due to its gene silencing by extensive promoter methylation [36,50,51], rendering them suitable models for expressing ALDH1L1 variants. To evaluate the impact of *ALDH1L1* SNPs on cellular metabotype, we expressed in both cell lines protein variants corresponding to ten major haplotypes found in human populations (Figure 1A). The expression was achieved by lentiviral transduction using the recombinant virus carrying both ALDH1L1 and GFP cDNA, each under the control of a different promoter [46]. This approach leads to co-expression of ALDH1L1 and GFP, allowing monitoring of transduced cells by fluorescence microscopy. Control cells were transduced with the virus carrying GFP cDNA only (no ALDH1L1 expression). In these experiments, cells exhibited comparable GFP fluorescence 96 h post-transduction for all used constructs, indicating efficient and equal viral delivery of different ALDH1L1 variants in both cell lines (Figure 2A,B). Quantification of total green fluorescence confirmed similar GFP expression levels in all groups (H0-9) and the GFP-expressing control (Figure 2A,B). Western blot assays further demonstrated robust ALDH1L1 expression for all haplotype variants (H0-H9) in both cell lines (Figure 2C,D). These conditions for ALDH1L1 expression were maintained for subsequent experiments.

### 3.2. Impact of ALDH1L1 SNP Variants on the Metabolome of A549 Cells

To assess metabotypes associated with ALDH1L1 haplotype variants (Figure 2A), we performed untargeted metabolomics. This analysis detected a total of 12,583 features, out of which 6327 metabolites were assigned (Supplementary Table S5). Features fitting the most confident assignment according to previously established classification [34,48] included 576 metabolites (OL1-PDA confidence rank, Supplementary Table S5), which were used for further analysis. Analysis of untargeted metabolome data using OPLS-DA showed that A549 cells expressing different ALDH1L1 haplotypes exhibited strong separation between the haplotype groups, indicating distinct metabotypes (Figure 3A). The OPLS-DA plot suggested the separation of the haplotypes into two clusters: one (designated as A549-GR1) included haplotypes (H3, H5, H6 and H8) clustered with the main (H0) haplotype, and the other (designated as A549-GR2) contained the rest of the haplotypes (H1, H2, H4, H7 and H9). While this clustering was exploratory, further comparison of the two groups (A549-GR1 and A549-GR2) using PCA and sPLSDA (Sparse Partial Least-Squares Discriminant Analysis) showed good group separation (Figure 3B,C), indicating that the two clusters reflect major metabolic differences between haplotypes. A heatmap of all metabolites showed that the two assigned groups separate clearly (Figure 3D). When two control groups were included in the analysis, parental A549 cells and A549 cells transduced for GFP expression (both groups do not have detectable ALDH1L1 expression), OPLS-DA indicated that the GFP control closely overlaps with the A549-GR2 cluster (Figure 3E). Overall, this analysis supports the classification of haplotype variants in two groups based on experimentally assessed metabotypes.

Further analysis was performed using three groups: A549-GR1, A549-GR2, and the control (cells expressing GFP only). PCA and sPLSDA for this three-group analysis confirmed close clustering of the control group lacking ALDH1L1 expression with A549-GR2 haplotypes (Figure 4A,B). We then performed pairwise comparisons amongst these three groups. As was expected from PCA/sPLSDA, large differences in metabolites between the A549-GR1 group and the two other groups were observed, with a total of 215 and 210 metabolites being significantly different in each comparison (Figure 4C). Cross-reference of significantly different metabolites from the two comparisons against the control (GFP) group showed that 48 identical metabolites were changed in response to either A549-GR2 or A549-GR1 haplotype expression (Figure 4D). Eight and 132 unique metabolites were altered in A549-GR2 and A549-GR1 groups, respectively (Figure 4D). Pathway analysis of metabolites differentiating A549-GR1 and A549-GR2 groups from the control group indicated that several pathways overlap between the two haplotype groups, but the extent of the effect was much stronger for the A549-GR1 group (Figure 4E–G). Most notably, these pathways include purine metabolism. Major non-overlapping pathways between A549-GR1 and A549-GR2 groups were (i) 1C and related pathways (we have assigned Gly/Ser/Thr and Cys/Met pathways as part of the 1C metabolism), (ii) TCA cycle and (iii) glutathione metabolism (Figure 4E–G). Of note, changes in 1C metabolism and the TCA cycle were characteristic of the A549-GR1 group, while changes in GSH metabolism were characteristic of the A549-GR2 group. Additional pathway analysis for metabolites overlapped between the A549-GR1 and A549-GR2 groups and were unique for the A549-GR1 group (Figure 4H,I), confirming 1C metabolism, TCA cycle, and GSH metabolism as major metabolic circuits that differentiated *ALDH1L1* haplotypes.

### 3.3. Metabolites and Pathways Affected by ALDH1L1 in A549 Cells

Analysis of specific metabolites from pathways that differentiated A549-GR1 and A549-GR2 showed a strong effect of ALDH1L1 on 5-methyltetrahydrofolate (5-methyl-THF; Figure S2), the stable reduced folate abundant in the cells. Of note, the levels of folic acid, the folate in the culture media, were only slightly different (Figure S2). High levels of p-aminobenzoic acid (Figure S2), the product of folate degradation [52], in the A549-GR1 group reflect high 5-methyl-THF. In addition, increased glycine and decreased serine in this group are in agreement with the regulation of serine to glycine conversion by ALDH1L1 [33]. The effect on TCA cycle intermediates of haplotypes, clustered with the main ALDH1L1 variant (A549-GR1 cluster), also coincides with previously reported findings [46]. The decrease in both reduced and oxidized glutathione (Figure S2), observed for the A549-GR1 cluster, indicates that the presence of fully functional ALDH1L1 affects glutathione biosynthesis rather than the use of GSH as an antioxidant. In agreement with this conclusion, the intermediate of cysteine biosynthesis, cystathionine, was strongly decreased in the A549-GR1 group (Figure S2). Insufficiency of glutathione biosynthesis is likely to evoke oxidative stress. Thus, the altered levels of several metabolites (cysteine-glutathione disulfide, methionine sulfoxide, methionine sulfone, oxoproline, and gamma-glutamylcysteine) observed in this group are in agreement with the effect of ALDH1L1 on glutathione metabolism. Overall, these data indicate that ALDH1L1 variants corresponding to certain haplotypes have altered regulatory capacity in cellular metabolism.

### 3.4. Effect of ALDH1L1 Variants on Metabolome of PC3 Cells

We have further analyzed and compared the metabolomes of another cell line, PC3, expressing the same *ALDH1L1* variants that were expressed in A549 cells (Figure 5). This analysis detected a total of 23,238 features, out of which 12,427 metabolites were assigned (Supplementary Table S6). Features fitting the most confident assignment according to previously established classification included 723 metabolites (OL1-PDA confidence rank, Supplementary Table S6), which were used for further analysis. The multivariate analysis (PCA) has shown clear separation of haplotypes in two clusters (Figure 5A). OPLS-DA and the heatmap demonstrated small within-group variance compared to between-group differences (Figure 5B,C). We have further analyzed these data by combining samples in two groups, which correspond to the separation of haplotypes in two clusters in both PCA and sPLSDA (Figure 5A,B). One group (designated as PC3-GR1) included haplotypes H0–H3, while the other group (designated as PC3-GR2) included haplotypes H4–H9. PCA comparing three groups (PC3-GR1, PC3-GR2 plus the GFP group as a negative control lacking ALDH1L1) showed good separation between the groups (Figure 5D). This analysis indicated that, in contrast to A549 cells, the metabolome of the PC3-GR1 cluster is more similar to the metabolome of the negative control, while the metabolome of the PC3-GR2 cluster is more different in PC3 cells (Figure 5C). Pairwise comparison showed that more metabolites differed between groups, confirming stronger dissimilarities between the control group and the PC3-GR2 cluster as compared to the PC3-GR1 cluster (Figure 5E). While exploratory, our group classification was helpful for the interpretation of the analysis of metabotypes linked to *ALDH1L1* genetic variants. Interestingly, the pathway analysis for two-group comparisons showed that 1C metabolism plays a very minor role in cellular response to ALDH1L1 in PC3 cells (Figure 5F–H).

### 3.5. Addressing Effect of ALDH1L1 in A549 vs. PC3 Cells

To further explore this unexpected phenomenon, we have compared the metabolites that are significantly different between the GR1 and GR2 clusters in A549 and PC3 cells using a Venn diagram (Figure 6A). The pathway analysis performed for each group of metabolites (unique for GR1 or GR2 clusters or overlapping between clusters) indicated that the effect of ALDH1L1 on 1C metabolism is a major differentiator between the two cell lines, A549 and PC3 (Figure 6B–D). To explain this difference, we examined in each cell line the levels of 5-methyltetrahydrofolate and folic acid. Since we ran experiments for each cell line independently, we did not perform direct cross-line comparisons but only within-the-line assessments of metabotypes and how they differ depending on *ALDH1L1* variants. We observed that the ratio between 5-methyl-THF (the active form of folate) and folic acid (the inactive form of folate) is shifted towards the latter in A549 cells lacking ALDH1L1 (Figure 6E). However, the expression of *ALDH1L1* variants corresponding to haplotypes from the GR1 cluster shifted the ratio towards the prevalence of 5-methyl-THF (Figure 6E). This effect did not take place in PC3 cells (Figure 6F). Interestingly, in A549 cells, ALDH1L1-associated changes in S-adenosylhomocysteine, one of the markers of folate status [53], closely followed the changes in 5-methyl-THF, which was not the case in PC3 cells (Figure 6E,F). In PC3 cells, however, ALDH1L1-associated accumulation of S-adenosylhomocysteine was accompanied by a decrease in methionine (Figure 6F). This effect was also apparent in A549 cells for the A549-GR1 haplotype cluster but not the A549-GR2 haplotype cluster (Figure 6E). These data are interpreted as cell-line-specific response patterns rather than direct quantitative comparisons of metabolite abundance between cell lines.

In PC3 cells, we were able to detect two additional folate species, 10-formyldihydrofolate (10-fDHF) and 5-formyltetrahydrofolate (5-fTHF, folinic acid) (Figure 6F). 10-fDHF, the product of oxidation of the ALDH1L1 substrate 10-formyl-THF, has been reported in the cell [54]. It is likely that 10-fDHF can be metabolized by ALDH1L1 to yield dihydrofolate, as was shown for two other 10-formyl-THF-utilizing enzymes, AICAR formyltransferase and methionyl-tRNA formyltransferase [54,55]. This conclusion is supported by almost complete depletion of 10-fDHF upon expression of the ALDH1L1 variant corresponding to the main (fully active) haplotype in PC3 cells (Figure 6F). Further, only partial depletion of 10-formyldihydrofolate in response to expression of certain haplotype variants (Figure 6F) indicates decreased functionality of *ALDH1L1* with corresponding SNPs. Another folate, 5-fTHF, which was detected in PC3 but not A549 cells (Figure 6F), is a storage form of reduced folate [56]. It can be converted by the enzyme MTHFS to 5,10-methenyl-THF [56], which in turn can be converted to 5,10-methylene-THF or 10-formyl-THF (Figure 6G). To explain differences in folate metabolism between A549 and PC3 cells, we evaluated mRNA levels of several cytosolic folate enzymes using the Protein Human Atlas portal. Observed significant differences between the two cell lines in mRNA levels for several key folate enzymes and transporters (Figure 6H) are in agreement with our metabolomic data (Figure 6E,F). Specifically, much higher levels of MTHFS mRNA in A549 cells might explain the fact that we did not detect 5-formyl-THF in these cells. In addition, larger differences in the expression of two key enzymes of glutathione metabolism, GSR and GCLC (Figure 6H), fit well with our findings that this pathway is among major metabolic discriminators of A549 and PC3 cells (Figure 6B).

### 3.6. A549 and PC3 Cells Have Drastically Different Metabotypes

Because of noticeable differences between effects of ALDH1L1 haplotypes on metabolomics of A549 vs. PC3 cells, we compared the overall metabotypes of the two cell lines. We ran samples from A549 and PC3 cells together in the same analytical run. A total of 21,550 peaks were recorded in this experiment, with 15,486 features assigned to specific metabolites. We then compared A549 and PC3 cells using a subset of the most confident metabolites (721 total). We observed strong separation of the groups by PCA (Figure S3A), which is also illustrated by a heatmap (Figure S3B). Of those 721 metabolites, 346 were significantly different between the two cell lines (FDR *p*-value below 0.05). Pathways differentiating A549 and PC3 cells included amino acid, nucleotide, 1C, and glutathione metabolism (Figure S3C). In addition to these pathways, strong differences in the levels of acyl-carnitines were observed between the two cell lines: with the 2-fold difference cutoff, 70 acylcarnitine species were significantly different between the two cell lines. Overall, our data showed a dramatic difference between metabotypes of A549 and PC3 cells, including 1C metabolism.

### 3.7. Effect of ALDH1L1 SNPs on Cell Viability, Colongenic Formation and Migration

Previous studies have demonstrated that ectopic expression of ALDH1L1 exerts strong antiproliferative and anti-migratory effects in several cell lines, including A549 and PC3 cells [46,51,57]. The present study investigated whether expression of *ALDH1L1* genetic variants produces similar effects on cell viability and migration, or if SNP-associated amino acid substitutions alter the protein regulatory capacity in the cell. *ALDH1L1* variants (H0–H9) were transduced at an MOI of 50 TU/cell into A549 and PC3 cells, and cell viability was monitored over time. Cell viability was assessed between 24 and 96 h time points across all groups using the CCK-8 assay. At 24 h, all groups displayed similar optical density (OD) values (approximately 0.2–0.3). By 96 h, the main ALDH1L1 haplotype (H0) exhibited the greatest reduction in CCK-8 signal consistent with stronger restriction of cell viability (Figure 7A,B). In A549 cells, all *ALDH1L1* genetic variants showed significantly increased proliferation at 96 h compared with the H0 variant (Figure 7A,B). A comparable trend was observed in PC3 cells. However, these cells were more sensitive to ALDH1L1 variants, and the effect of most variants on CCK-8 signal was more similar to the effect of the main enzyme haplotype (H0) (Figure 7A,B).

We then performed a colony-formation assay, which provides an independent assessment of clonogenic survival and long-term proliferative capacity (14 days) (Figure 7C,D). In both cell lines, all genetic variants suppressed colony formation to varying degrees. The main ALDH1L1 haplotype (H0) produced the greatest reduction in colony number (Figure 7C,D, right panel). Notably, there were distinct differences in colony size between control and ALDH1L1-expressing cells (Figure S4A,B). Control cells formed large, dense colonies, whereas ALDH1L1-expressing cells produced significantly smaller and less compact colonies. This reduction in colony size suggests that ALDH1L1 impairs long-term proliferative potential and clonogenic survival in both cell lines. A similar effect was observed with the other variants (H1–H9), further supporting their suppressive impact on long-term proliferation (Figure 7C,D) compared to ALDH1L1-deficient control cells. However, the magnitude of suppression varied among the variants and was generally less pronounced than that observed with the main haplotype (H0) (Figure 7C,D).

Next, we assessed the migratory capacity of ALDH1L1-expressing cells. A549 and PC3 cells carrying *ALDH1L1* genetic variants exhibited significantly reduced ability to suppress migration compared to cells expressing the main haplotype (Figure 8A and Figure 9A). Variations in the percentage of wound closure were observed among the genetic variants in both cell lines. As expected, control cells lacking ALDH1L1 expression exhibited nearly complete wound closure at 48 h (Figure 8A and Figure 9A). Previous studies from our lab have demonstrated that ALDH1L1 suppresses motility of A549 cells by promoting cofilin dephosphorylation and remodeling of the actin cytoskeleton [58]. To assess whether *ALDH1L1* genetic variants retain this regulatory function, we examined levels of phosphorylated and total cofilin in *ALDH1L1* variant-expressing A549 and PC3 cells (Figure 8B and Figure 9B). In A549 cells, expression of all ALDH1L1 variants led to a significant decrease in phosphorylated cofilin (Figure 8B). Of note, the H0, H1 and H5 variants showed nearly undetectable levels of phosphorylated cofilin (Figure 8B). In contrast, PC3 cells did not exhibit a similar pattern; although phosphorylated cofilin levels were reduced compared to control cells, they remained relatively elevated across all samples in PC3 cells (Figure 9B). These findings suggest that, in PC3 cells, alternative signaling mechanisms may partially offset ALDH1L1-mediated regulation and contribute to the reduced migratory capacity observed in ALDH1L1-expressing cells.

## 4. Discussion

ALDH1L1 is a common enzyme in folate metabolism with a very high expression in the liver and several other tissues [50]. It has been calculated that in hepatocytes it represents about 1% of the total cytosolic protein [29]. At the same time, ALDH1L1 is typically not expressed in proliferating cells, with examples including many cancer cell lines and human skin fibroblasts [36,50,59,60]. In agreement with this phenomenon, re-expression of the enzyme in different types of cancer cells inhibits proliferation and induces cell death [30]. While ALDH1L1 expression in cancer cells causes the activation of several signaling pathways [31,51,58,61,62], the primary effect of ALDH1L1 is on the folate cycle and related metabolic pathways. In fact, the inhibitory effect of ALDH1L1 in cancer cells can be rescued by high supplementation of reduced folate, folinic acid, or purine nucleotide precursor, hypoxanthine [50,63]. This explains the strong expression of the enzyme in regenerating livers since the liver has high levels of reduced folate and purine nucleotides [50].

The enzymatic role of ALDH1L1 is well established: the enzyme converts 10-formyl-THF to THF, simultaneously generating NADPH and oxidizing the folate-bound 1C group to CO_2_ [29]. This reaction is linked to several metabolic circuits in the cell. (i) It competes with two reactions of de novo purine biosynthesis for the same substrate, 10-formyl-THF, thus controlling its flux to this pathway. (ii) It regenerates THF, the coenzyme central to folate metabolism, since this is the only 1C group acceptor in the reduced folate pool. In support of the importance of this role, knockout of *ALDH1L1* in mice not only shifts the ratio between 10-formyl-THF and THF but also shifts the ratio between serine and glycine due to insufficient THF levels [33]. (iii) The contribution of ALDH1L1 to NADPH generation could be important, as was demonstrated for its mitochondrial paralog [3]. In fact, it has been recently reported that ALDH1L1-generated NADPH maintains mitochondrial redox homeostasis [64] while downregulation of the enzyme significantly inhibited the occurrence of NADP^+^-dependent reactions [61]. (iv) It has also been shown that the enzyme indirectly affects oxidative phosphorylation [62]. (v) Overall, ALDH1L1 controls the level of 1C groups in the reduced folate pool as it irreversibly removes them from the pool as CO_2_. This also affects the methylation capacity of the cell [32] and is linked to regulation of cellular motility through the actin-cofilin axis [58]. Due to such key roles, the protein is an important regulator of the cellular metabolic program.

Notably, *ALDH1L1* shows a high frequency of non-synonymous exonic SNPs at the polymorphic loci rs3796191, rs2886059, rs9282691, rs2276724, rs1127717, and rs4646750 [18]. These SNPs cause amino acid substitutions within each of the three ALDH1L1 domains (Figure 1). Two of these substitutions involve amino acid residues strictly conserved among all species, and one is conserved in all but Zebrafish (Figure 1). Such conservation typically indicates structurally or functionally important residues whose substitution could have effects on enzyme activity, protein folding/stability, or interaction with other proteins [65,66]. Potential effects of SNP amino acid substitutions on ALDH1L1 functionality have been previously discussed [18]. Complex organization of ALDH1L1 [67] indicates that such substitutions might not only affect enzymatic activity through direct interactions with substrate or coenzymes but also by interfering with the functional tetramer assembly, restricting domain communication, or diminishing the attachment of the prosthetic group. Our goal in this study was to evaluate the general effect of *ALDH1L1* variants on cellular metabolism and proliferation to provide knowledge of whether individuals with specific SNPs might have altered ALDH1L1-linked metabolic regulation, which could be involved in the etiology of certain metabolic disease. The differential role of the enzyme polymorphic variants in metabolic regulation was supported by genome-wide association studies (GWAS), which showed that SNPs in ALDH1L1 are associated with the serum serine-to-glycine ratio [68]. Further targeted analysis of five exonic non-synonymous *ALDH1L1* SNPs in a cohort of Hispanic children not only confirmed a strong association between these SNPs and serum serine-glycine ratio but also suggested their link to metabolic disease risk [69].

Genomic data showed that several non-synonymous *ALDH1L1* SNPs are inherited together, thus producing specific *ALDH1L1* haplotype variants [18]. Half of the most common *ALDH1L1* variants are represented by haplotypes with two or three SNPs (Figure 1, top panel). However, SNPs are commonly analyzed one at a time, the approach not fully reflecting their functional effects due to the lack of consideration of the combined effect of the SNPs. Therefore, our study investigated *ALDH1L1* haplotypic variants rather than SNP variants. Previous analysis of cells expressing the *ALDH1L1* variant corresponding to the main haplotype vs. ALDH1L1-deficient cells identified metabolic pathways under the control of the enzyme, which include 1C, nucleotide and amino acid metabolism, glutathione biosynthesis, and TCA cycle, pathways crucial for cellular proliferation [34,47]. Findings in the present study showed that the effects of some *ALDH1L1* haplotype variants on these pathways are weaker than the effect of the main haplotype. In addition, certain common variants were much less effective in the control of cellular proliferation. Such metabolic and functional consequences of *ALDH1L1* non-synonymous exonic SNPs for the cell raise the question regarding the biological advantage of the selection of these enzyme variants. Though the presence of ALDH1L1 variants likely alters intracellular folate homeostasis, such an effect could be beneficial depending on folate availability. For example, under conditions of low dietary folate or low input of 1C groups, specific *ALDH1L1* haplotypes might favor the retention of such groups.

Our study also demonstrated that *ALDH1L1* haplotype variants inhibit proliferation, colony formation, and migration to a lesser extent than the main haplotype. Of note, there were noticeable variations in inhibitory effects between these variants and across both cell lines. Interestingly, though, while the haplotype-associated metabotypes can be segregated into two major clusters in both studied cell lines, cell proliferation and migration did not clearly follow this trend. Apparently, global similarity in metabolomic profiles does not necessarily predict identical effects on cellular behavior. One explanation for this phenomenon could be additional functions of ALDH1L1, not captured by our study, which are differentially affected by haplotype variants. One of these functions could be the direct binding and protection from degradation of unstable reduced folate coenzymes [40]. Another could be associated with NADPH generation [61], a function not assessed in our study. Finally, the interaction of ALDH1L1 with other proteins, suggested by the literature [39,64,70,71,72,73], could contribute to the effect on cellular phenotype independently of the metabolic role of the enzyme. Of note, functions of several folate enzymes, linked to their interactions with other proteins, have been reported [74,75,76,77]. In addition, a recent study reported the complex formation between ALDH1L1 and glutamine synthetase, leading to the activation of the latter enzyme and enhanced glutamate clearance [62]. A deeper analysis of correlations and associations between variant-associated metabotypes and cellular phenotype is required to address the role of ALDH1L1 beyond its catalytic function. Notably, the grouping of haplotypes into GR1 and GR2 was derived from supervised methods and was therefore data-driven. Because of this, these groupings should be considered exploratory and should be further validated in future experiments.

It is likely that the consequences of *ALDH1L1* variants can be modified by the overall metabolic and signaling landscape of specific organs or cell types. In this regard, our study demonstrated strong difference in metabotypes between A549 and PC3 cells, which might contribute to different metabolic responses of these cells to the haplotype variants. The outcome of the *ALDH1L1*-linked regulation largely depends on the overall capacity of the folate cycle, as was discussed above for the liver. Another example of such dependence was the study demonstrating that A549 cells can tolerate the ALDH1L1 antiproliferative effect under conditions of high folate supplementation and increased expression of DHFR, the key enzyme incorporating folic acid into the reduced folate pool [63]. Specifically relevant to cancer cells, the downstream cellular responses to ALDH1L1’s effect on the folate cycle involve different signaling proteins, which could further alter cellular metabolism. One such protein downstream of *ALDH1L1* is the tumor suppressor p53, which has been linked to the regulation of key metabolic pathways [78]. While A549 cells have wild-type p53, PC3 cells have lost p53, and their response to ALDH1L1 activates different signaling mechanisms [51,79]. Further, A549 cells harbor *KRAS* and *STK11/LKB1* mutations in contrast to PC3 cells, in which these genes are not mutated [80]. Of note, links between *ALDH1L1* and *KRAS* [81] or the key LKB1 target AMPK [35] have been reported. Overall, different genetic backgrounds could be a strong contributing factor differentiating metabolic responses to *ALDH1L1* variants in A549 vs. PC3 cells.

## 5. Conclusions

Based on our data, we offer the following conclusions regarding the role of *ALDH1L1* haplotypes. (1) The advantage of our approach in this study was that instead of exploring the roles of separate SNPs, we focused on the effect of haplotype variants, which is more biologically relevant. (2) *ALDH1L1* haplotype variants are associated with different metabolic effects in the cell. (3) These variants mainly impact the same metabolic pathways but exert a different extent of regulatory effect. (4) While the primary impact of ALDH1L1 is on 1C metabolism, additional pathways under the regulation of the enzyme include amino acid, purine, and glutathione metabolism as well as the TCA cycle. (5) The effect of the haplotype variants is likely cell type/tissue-specific. (6) All studied haplotype variants have diminished capacity to regulate cell proliferation and migration as compared to the main haplotype, although they still produced noticeable inhibitory effects compared to ALDH1L1-deficient cells. Of note, the magnitude of these effects was different between haplotype variants. Future analysis of human cohorts should provide insight into the role of *ALDH1L1* haplotype variants in the regulation of metabolism at the whole organism level and explore links between the haplotypes and metabolic diseases. Such analysis will also address the question of whether certain *ALDH1L1* haplotype variants affect the risk of tumorigenesis and whether such an effect is organ-specific.

## Figures and Tables

**Figure 1:**
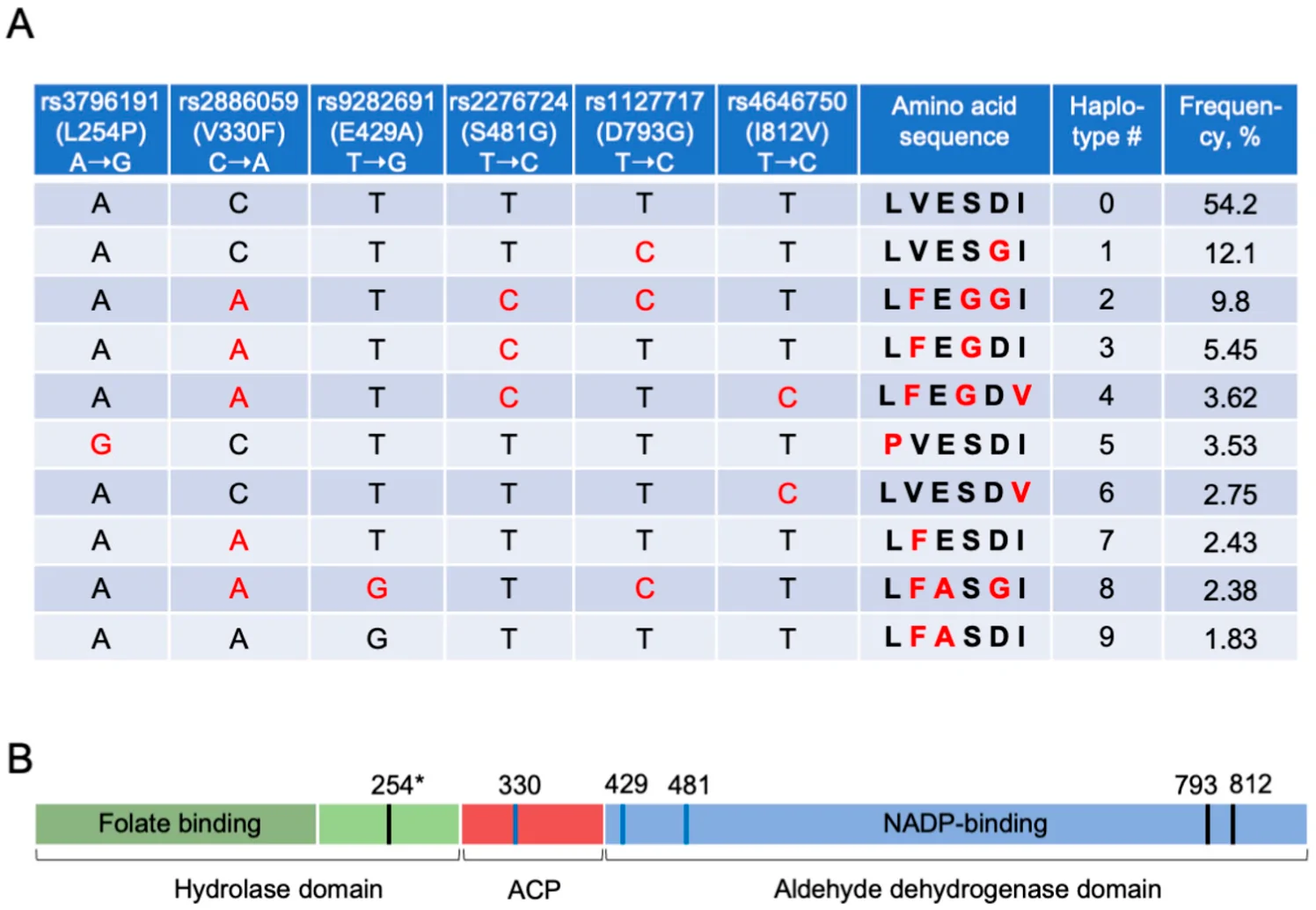
Common exonic SNPs within the *ALDH1L1* gene. (**A**) Table listing six non-synonymous ALDH1L1 exonic SNPs, corresponding nucleotide and amino acid substitutions, and the ten most common haplotypes in humans associated with these SNPs. (**B**) Location of residues affected by non-synonymous SNPs in the ALDH1L1 amino acid sequence. Three ALDH1L1 functional domains are indicated by green, red, and blue boxes. Residues 793 and 812 are conserved among all species; (*) residue 254 is conserved in all species except Zebrafish. Haplotype numbering is based on the ranking from highest to lowest frequency (derived using UCSC Genome Browser; https://genome.ucsc.edu; accessed on 1 October 2026).

**Figure 2:**
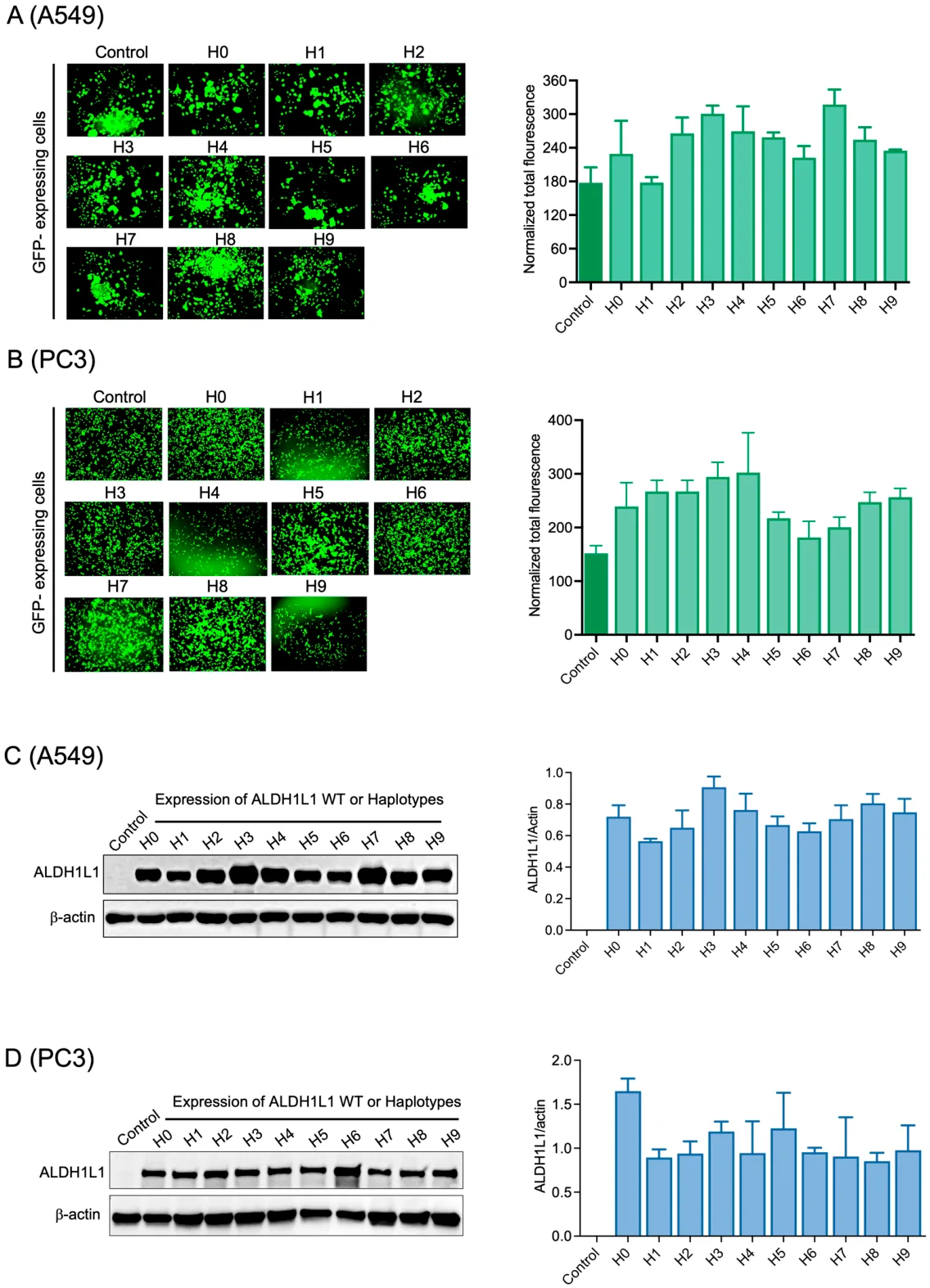
Expression of ALDH1L1 WT or genetic variants in A549 and PC3 cells. (**A**,**B**) Left panel, representative images of A549 and PC3 cells at day 4 post-transduction with a control lentiviral vector expressing GFP alone or with a lentiviral vector expressing GFP and ALDH1L1 (WT or SNP variants) at a multiplicity of infection (MOI) of 50 TU/cell. Right panel, bar graph showing the mean + SEM of total GFP fluorescence intensity normalized to image area (*n* = 3). Statistical differences were assessed using one-way ANOVA comparing all groups. No significant differences were observed between control and ALDH1L1-expressing groups. (**C**,**D**) Left panel, immunoblot analysis of A549 and PC3 cells (*n* = 3) transduced with either the control vector or vectors harboring ALDH1L1 WT or each variant at an MOI of 50 TU/cell for 4 days. Right panel, bar graph showing the mean ± SEM of ALDH1L1 band intensities normalized to β-actin levels. Statistical differences were assessed using one-way ANOVA comparing all groups. No significant differences were observed among groups. Uncropped and unmodified immunoblot images corresponding to (**C**,**D**) left panel, are shown in Figure S1A,B.

**Figure 3:**
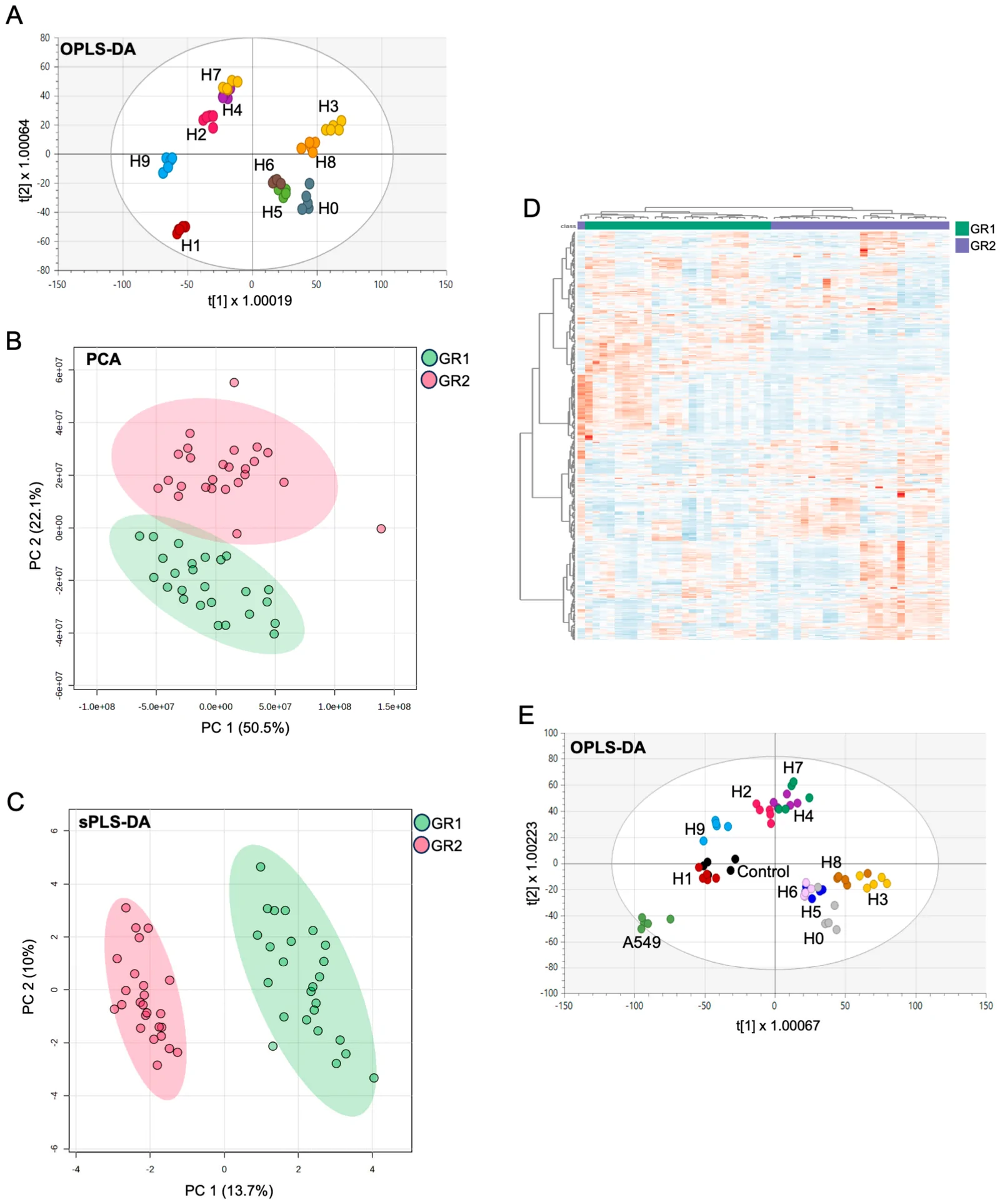
*ALDH1L1* genetic variants cause different metabotypes in A549 cells. (**A**). OPLS-DA for the comparison of metabotypes corresponding to 10 major human *ALDH1L1* haplotypes; haplotype numbering as in Figure 1A. (**B**,**C**) PCA and sPLSDA for the comparison of haplotype-associated metabolomics assigned in two groups (A549-GR1, main ALDH1L1 variant-clustered haplotypes; A549-GR2, ALDH1L1-deficient group-clustered haplotypes). (**D**). Heatmap for the two-group analysis. (**E**). OPLS-DA for the comparison of metabotypes corresponding to the 10 most common *ALDH1L1* haplotypes and metabotypes of two ALDH1L1-deficient cells, parental A549 (A549 group) and A549 transduced for GFP expression (GFP group). Haplotype numbering as in panels (**A**,**B**). For each haplotype variant, five biological replicates were analyzed.

**Figure 4:**
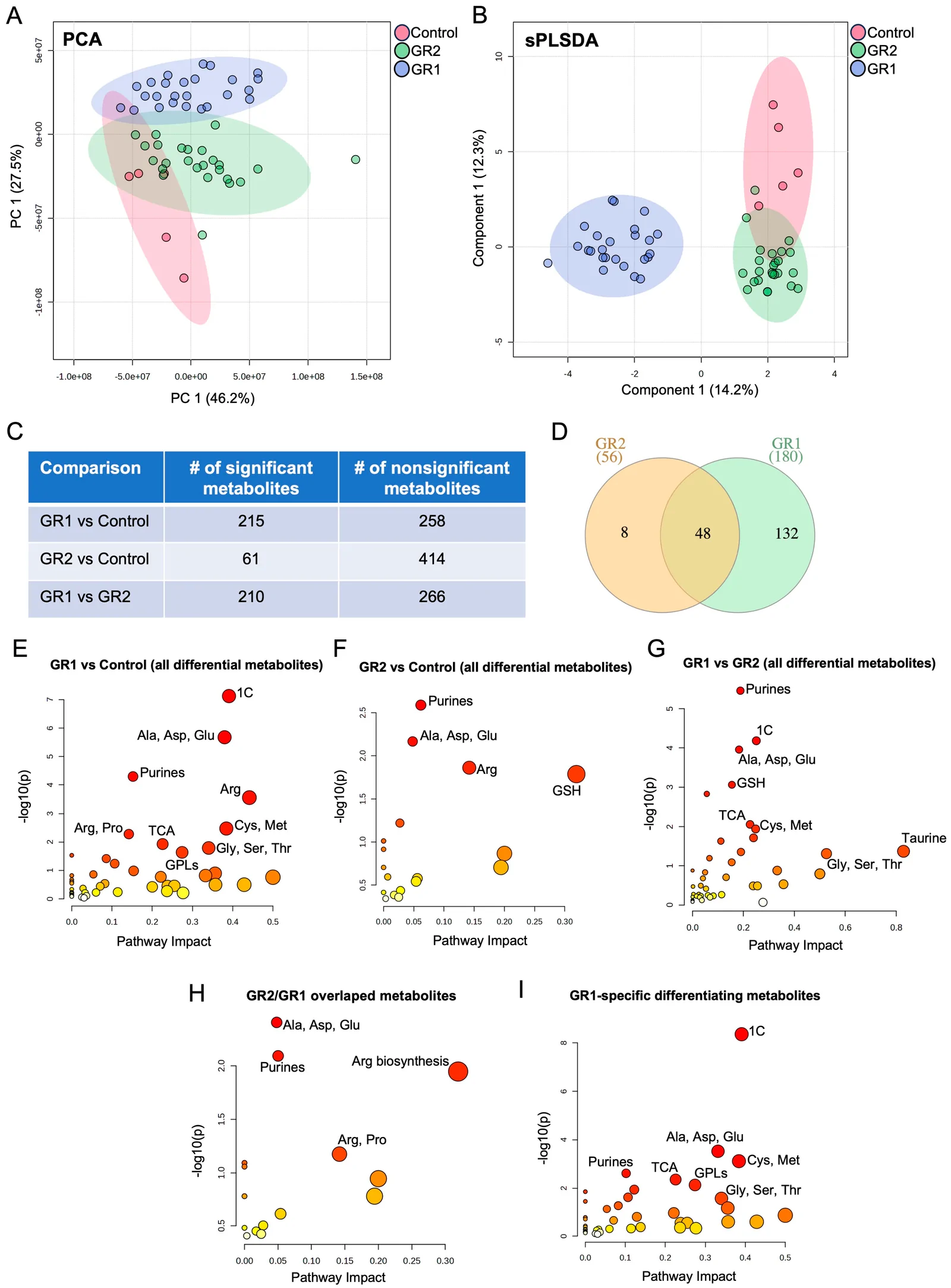
Analysis of A549 metabotypes within two *ALDH1L1* haplotype clusters. (**A**,**B**) PCA and sPLSDA of *ALDH1L1* haplotype-associated metabolomics assigned in two groups as in Figure 1 in comparison with the negative control (GFP-expressing group). (**C**) Number of significant (FDR *p* < 0.05) and non-significant metabolites in pairwise comparison between groups from (**A**,**B**). (**D**) Venn diagram showing the number of common and unique metabolites changed in two *ALDH1L1* haplotype clusters compared to the *ALDH1L1* negative control (GFP). (**E**–**I**) Pathway analyses for the indicated group comparison using metabolites with a nominal *p*-value < 0.05. For each haplotype variant, five biological replicates were analyzed. Pathways are plotted according to significance (y-axis) and pathway impact value (x-axis). Larger circles represent greater pathway enrichment; red color indicates higher statistical significance.

**Figure 5:**
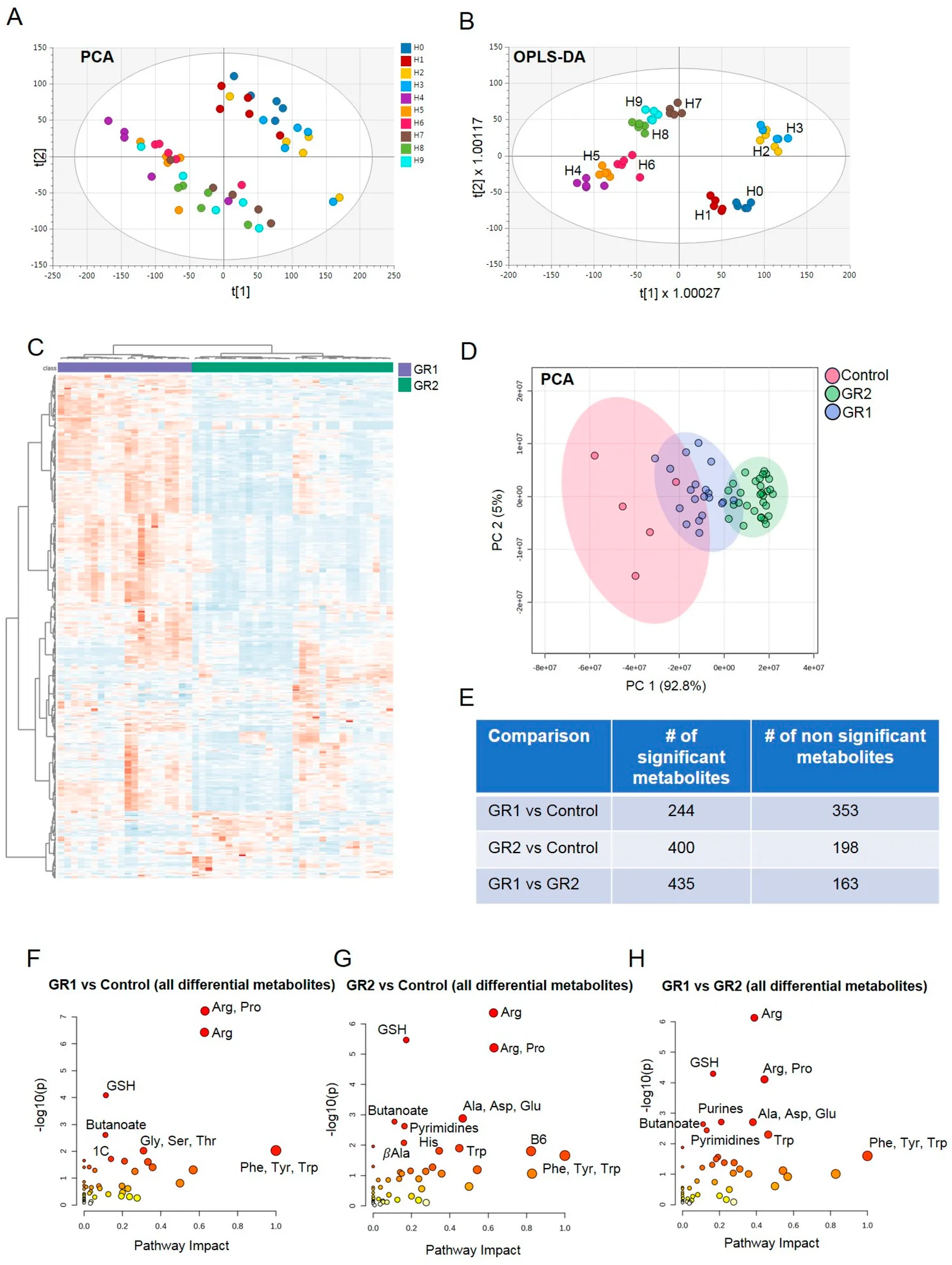
Analysis of metabolomes associated with *ALDH1L1* haplotype variants in PC3 cells. (**A**) PCA and (**B**) OPLS-DA for the comparison of metabotypes corresponding to 10 major human *ALDH1L1* haplotypes; haplotype numbering as in Figure 1A. (**C**) Heatmap for the two-group analysis. (**D**) PCA of *ALDH1L1* haplotype-associated metabolomics assigned in two groups (PC3-GR1, main *ALDH1L1* variant-clustered haplotypes; PC3-GR2, the second haplotype cluster) in comparison with the negative control (GFP-expressing group). (**E**) Number of significant (FDR *p* < 0.05) and non-significant metabolites in pairwise comparison between groups from (**D**). (**F**–**H**) Pathway analyses for the indicated group comparison using metabolites with a nominal *p*-value < 0.05. For each haplotype variant, five biological replicates were analyzed. Pathways are plotted according to significance (y-axis) and pathway impact value (x-axis). Larger circles represent greater pathway enrichment; red color indicates higher statistical significance.

**Figure 6:**
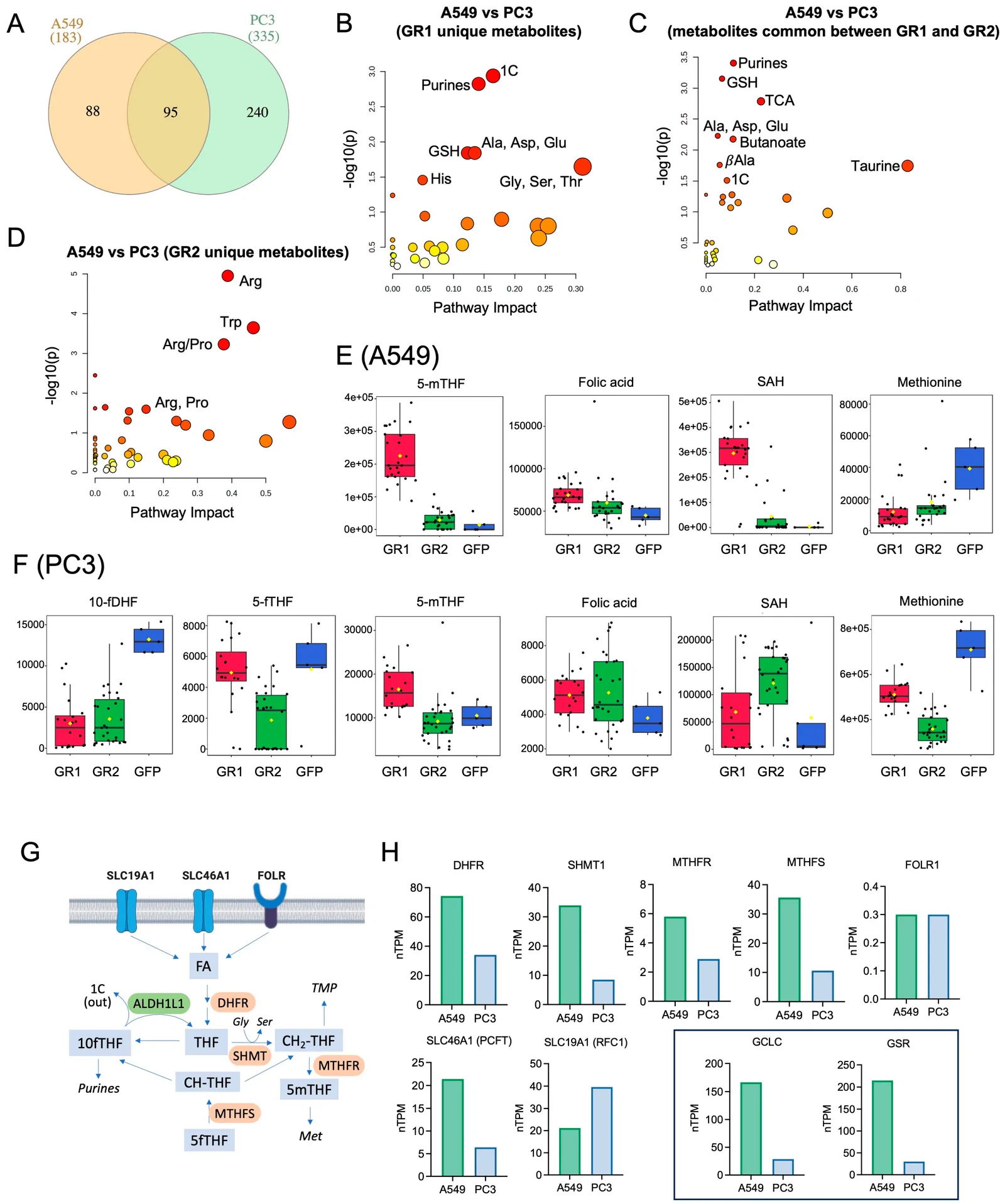
Comparison of ALDH1L1-associated metabotype responses in A549 and PC3 cells. (**A**) Venn diagram showing the comparison between A549 and PC3 cells for the number of common and unique significant metabolites differentiating GR1 and GR2 haplotype clusters. (**B**–**D**) Pathway analyses for the indicated groups from (**A**). Pathways are plotted according to significance (y-axis) and pathway impact value (x-axis). Larger circles represent greater pathway enrichment; red color indicates higher statistical significance. (**E**) Relative within-run peak intensities of selected folate-related metabolites in A549 cells. (**F**) Relative within-run peak intensities of the corresponding metabolites in PC3 cells (note: 10-formyldihydrofolate (10-fTHF) and 5-formyltetrahydrofolate (5-fTHF) were not detected in A549 cells). The *Y*-axis in (**E**,**F**) indicates peak intensities. (**G**) Schematic depicting cytosolic folate pathways; three folate transporters (SLC19A1, SLC46A1 and FOLR1) are shown. (**H**) Levels of mRNA for proteins indicated in (**G**) in A549 vs. PC3 cells (taken from the Human Protein Atlas; www.proteinatlas.org). Levels of two key enzymes of glutathione (GSH) metabolism are shown in the box. A549 and PC3 samples were evaluated in separate analytical runs without a shared pooled QC sample or cross-run batch correction; peak intensities in panels E and F are intended to illustrate within-cell-line response patterns. Abbreviations are as follows: SAH, S-adenosylhomocysteine; THF, tetrahydrofolate; 10fTHF, 10-formyltetrahydrofolate; CH-THF, methenyltetrahydrofolate; 5fTHF, 5-formyltetrahydrofolate; 5mTHF, 5-methyltetrahydrofolate; CH2-THF, methylene-tatreahydrofolate; DHFR, dihydrofolate reductase; SHMT, serine hydroxymethyltransferase; MTHFS, 5,10-methenyltetrahydrofolate synthase; MTHFR, methylenetetrahydrofolate reductase; Met, methionine; TMP, thymidine monophosphate; Gly, glycine; Ser, serine; GCLC, glutamate-cysteine ligase catalytic subunit; GSR, glutathione reductase.

**Figure 7:**
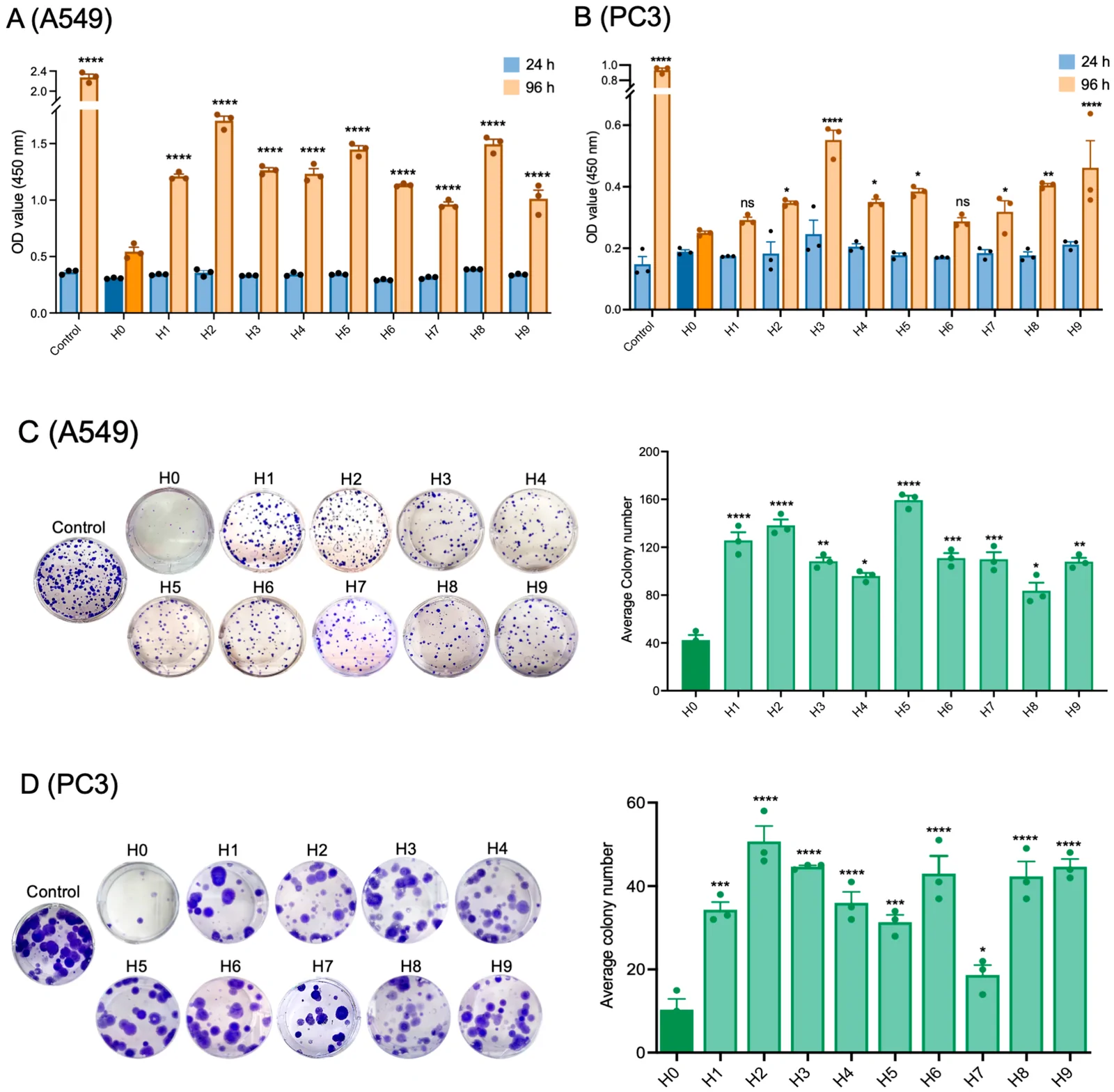
Effect of *ALDH1L1* genetic variants on A549 and PC3 cell proliferation and colony formation. Proliferation of (**A**) A549 and (**B**) PC3 cells expressing lentivirus-delivered *ALDH1L1*-WT or -SNP variants following transduction at an MOI of 50 TU/cell, assessed by CCK-8 assay. (**C**) Left panel, representative images of colonies formed by A549 cells (1 × 10^3^) transduced with vectors expressing *ALDH1L1*-WT or -SNP variants at an MOI of 50 TU/cell. Right panel, bar graph showing the mean colony numbers ± SEM from three independent experiments. (**D**) Left panel, representative images of colonies formed by PC3 cells (1 × 10^3^) transduced with vectors expressing *ALDH1L1*-WT or -SNP variants at an MOI of 50 TU/cell. Right panel, bar graph showing the mean colony numbers ± SEM from three independent experiments. Statistical significance was assessed by one-way ANOVA followed by multiple-comparison testing, comparing each *ALDH1L1* variant with H0: **** *p* < 0.0001, *** *p* < 0.001, ** *p* < 0.01, * *p* < 0.05, ns = not significant.

**Figure 8:**
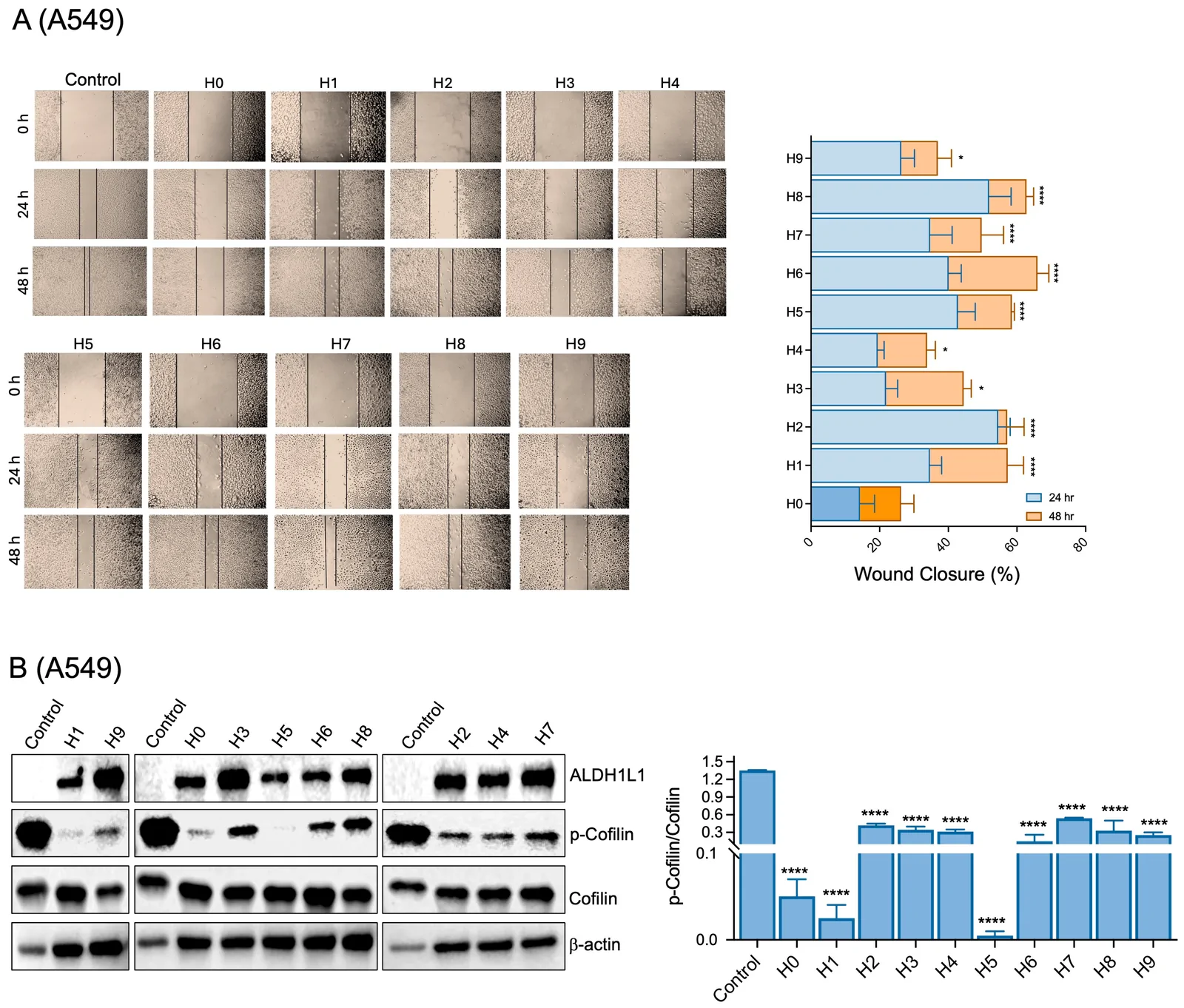
Effect of *ALDH1L1* variants on A549 cell migration. (**A**) On the left, wound-healing assay representative images for A549 cells expressing ALDH1L1-WT or corresponding variants. Black lines indicate the cell-free area. Right, bar graph showing the mean ± SEM of wound closure (*n* = 3) at 24 and 48 h post-scratch from three independent biological experiments. Statistical significance was determined by one-way ANOVA followed by Tukey’s multiple comparison test, with comparisons restricted to each *ALDH1L1* variant versus H0: **** *p* < 0.0001, * *p* < 0.05. (**B**) Left, representative immunoblot images of p-cofilin and cofilin from A549 cells (*n* = 3) transduced with either the control vector or vectors harboring ALDH1L1 WT or each of the genetic variants at an MOI of 50 TU/cell. Right panel: Intensity of the phospho-cofilin band was normalized to the corresponding total cofilin band from the same biological sample. The resulting phospho-cofilin/total cofilin ratio was calculated independently for each biological replicate (*n* = 3) and used for statistical analysis. Data are presented as mean ± SEM. Statistical significance was assessed by one-way ANOVA followed by multiple-comparison testing, comparing each ALDH1L1 variant with control (GFP). Uncropped and unmodified immunoblot images corresponding to panel B are shown in Figure S5.

**Figure 9:**
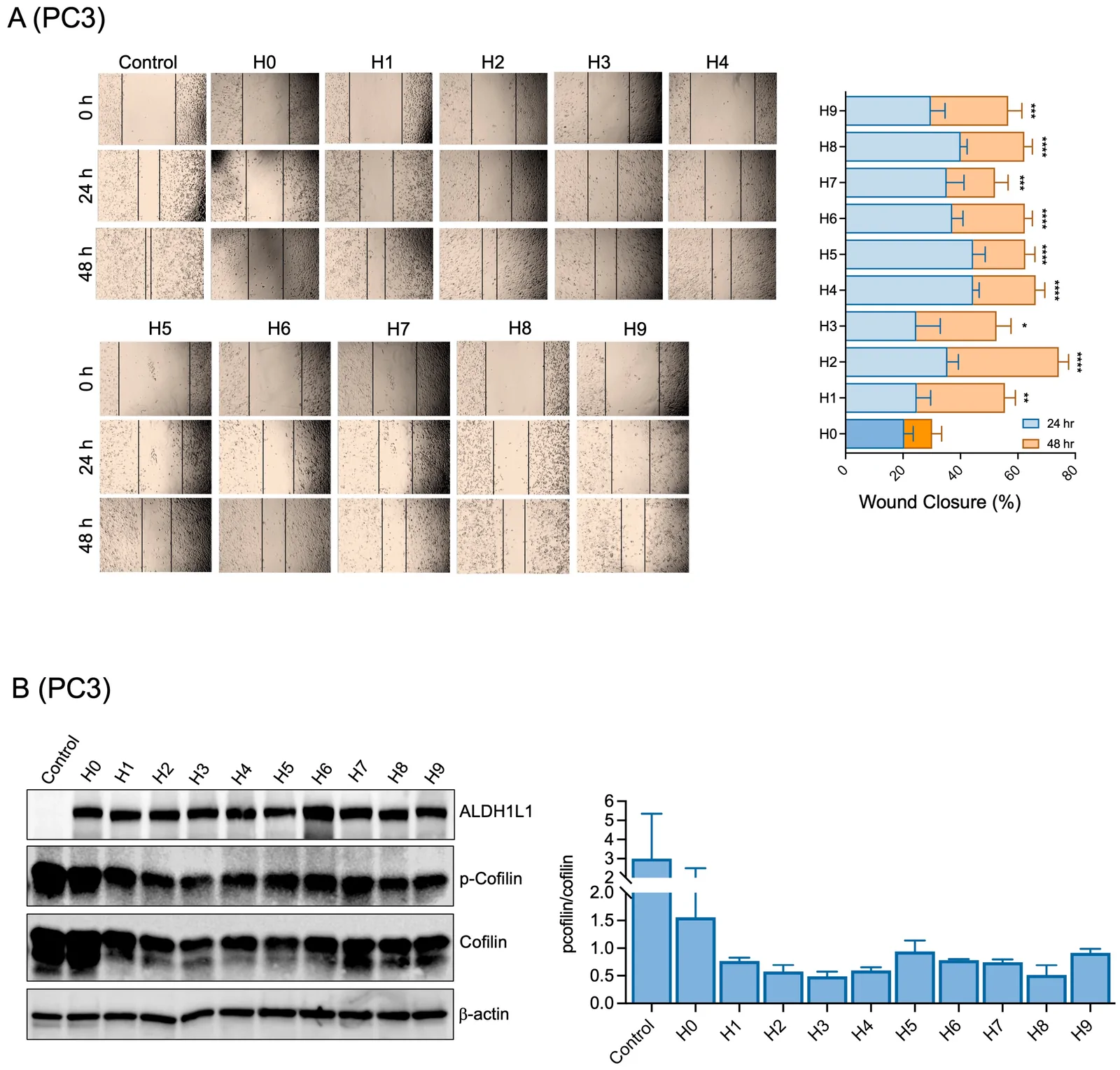
Effect of *ALDH1L1* genetic variation on PC3 cells migration. (**A**) Representative images of wound-healing assays for ten haplotypes (Left, black lines indicate the cell-free areas), with bar graphs on the right showing % of wound closure as means ± SEM (*n* = 3) at 24 and 48 h post-scratch from three independent biological experiments. Statistical significance analyzed by one-way ANOVA followed by Tukey’s multiple comparison test, with comparisons restricted to each *ALDH1L1* variant versus H0: **** *p* < 0.0001, *** *p* < 0.001, ** *p* < 0.01, * *p* < 0.05. (**B**) Left, representative immunoblot images of ALDH1L1, p-cofilin and cofilin from A549 cells (*n* = 3) transduced with the control vector or each of the vectors harboring *ALDH1L1* WT, and the genetic variants, at an MOI of 50 TU/cell. Right panel: quantification of the p-cofilin/cofilin ratio from the immunoblot images. Intensity of the phospho-cofilin band was normalized to the corresponding total cofilin band from the same biological sample. The resulting phospho-cofilin/total cofilin ratio was calculated independently for each biological replicate (*n* = 3) and used for statistical analysis. Data are presented as mean ± SEM. Statistical significance was assessed by one-way ANOVA followed by multiple-comparison testing, comparing each ALDH1L1 variant with the control (GFP). No statistically significant differences were observed between any ALDH1L1 variant and the control (GFP). Uncropped, unmodified immunoblot images corresponding to panel B are shown in Figure S6.

## Data Availability

The data presented in this study are openly available in Metabolomics Workbench at https://dev.metabolomicsworkbench.org:22222/data/DRCCMetadata.php?Mode=Study&StudyID=ST004743&Access=NxwB1283, [datatrack_id:7342 study_id:ST004743], and https://dev.metabolomicsworkbench.org:22222/data/DRCCMetadata.php?Mode=Study&StudyID=ST004744&Access=OhvH7178, [datatrack_id:7343 study_id:ST004744]. Both data accessed on 9 April 2026.

## Supplementary Materials

The following supporting information can be downloaded at: https://www.mdpi.com/article/10.3390/cells15212026/s1, Figure S1: Original immunoblot images corresponding to Figure 2C (A) and Figure 2D (B). St, Standard protein ladder, Figure S2: Comparison of metabolites from three pathways most significantly differentiating WT and GR2 groups in A549 cells, Figure S3: A549 and PC3 cells have very different metabotypes. (A) PCA for the comparison of untargeted metabolomics between A549 and PC3 cells based on 721 most confident metabolites. (B) Heat map for 125 top metabolites different between A549 and PC3 cells. (C) Pathways differentiating A549 and PC3 cells. The analysis was performed using MetaboAnalyst 6.0. For each cell line, six biological replicates were analyzed, Figure S4: Representative images of the colongenic assay of control and ALDH1L1-expressing (A) A549 and (B) PC3 cells. All images were taken using a Keyence BZ-X700 microscope, Figure S5: Original immunoblot images corresponding to Figure 8B. St, Standard protein ladder, Figure S6: Original immunoblot images corresponding to Figure 9B. St, Standard protein ladder; Table S1: ALDH1L1 genetic variants identified across the NCI-60 cancer cell line panel using the CellMiner database, Table S2: Genetic variants (SNPs) in ALDH1L1 across tested cancer cell lines, Table S3: Primer sequences used for DNA mutagenesis for all ALDH1L1 SNP variants, Table S4: Statistical parameters for OPLS-DA models, Table S5: Untargeted metabolomics dataset for A549 cells, Table S6: Untargeted metabolomics dataset for PC3 cells.
