# Supplementary material for: Genetic Variations in the Folate Regulatory Enzyme ALDH1L1: Impact on Cell Metabolism, Proliferation, and Motility
Source: Cells. 2026 Nov 6;15(21):2026. doi: 10.3390/cells15212026 (PMC13761728; doi:10.3390/cells15212026)
Supplement: Supplementary file 1 [file cells-15-02026-s001.zip › Revised Supporting Figure_110526.pdf]

Supplementary Figure S1

A. A549

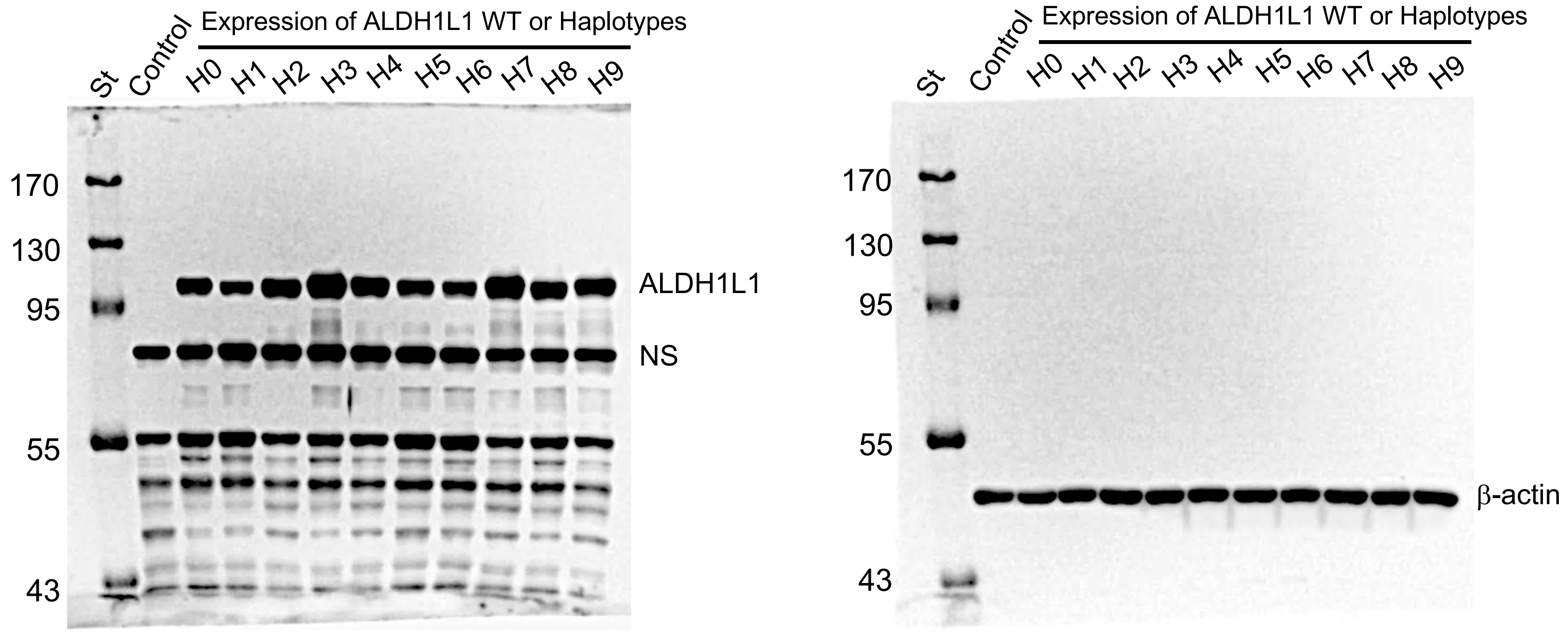

B. PC3

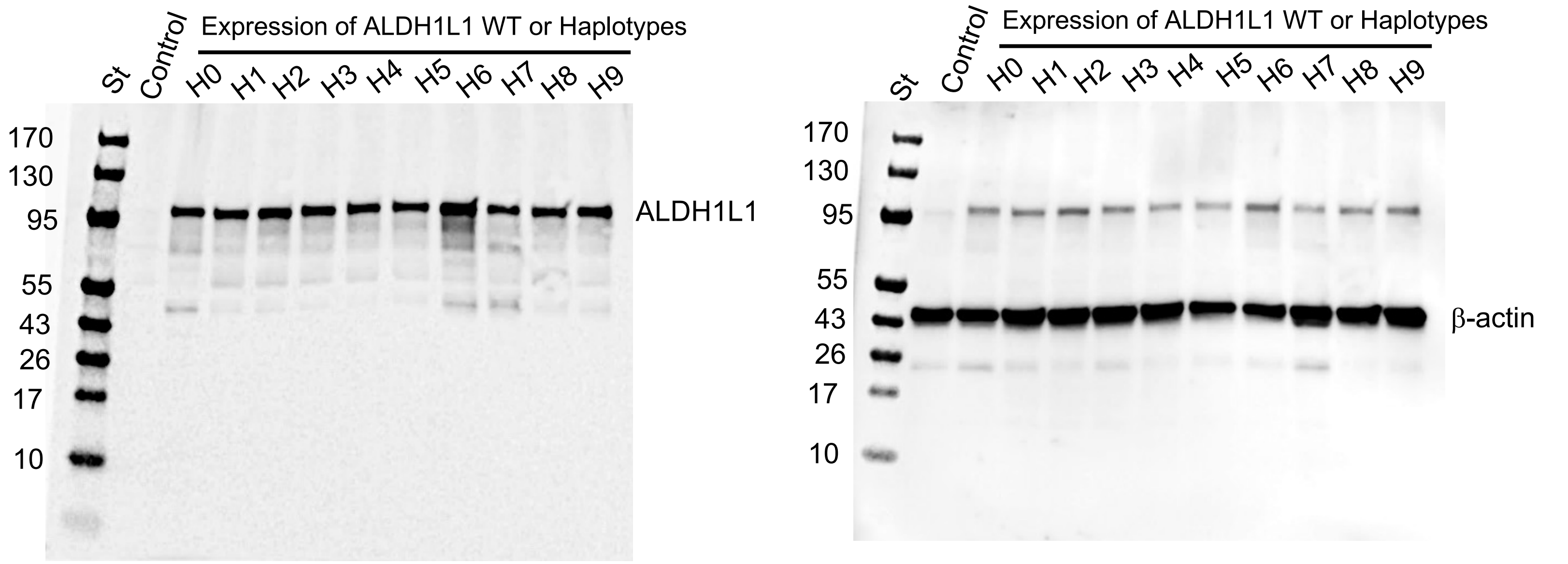

Figure S1. Original immunoblot images corresponding to Figure 2C (**A**) and Figure 2D (**B**). St, Standard protein ladder.

Supplementary Figure S2

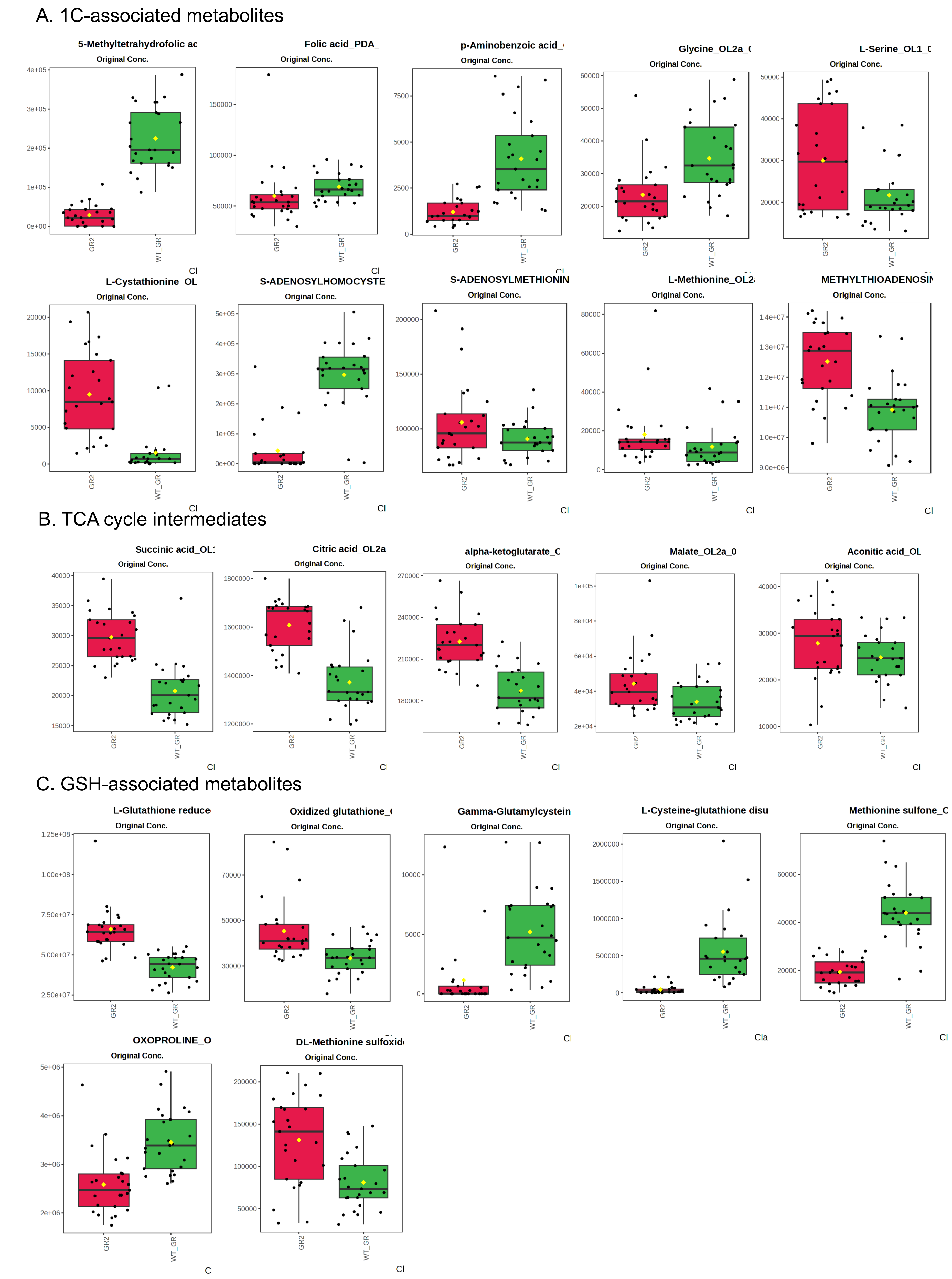

Figure S2. Comparison of metabolites from three pathways most significantly differentiating WT and GR2 groups in A549 cells.

Supplementary Figure S3

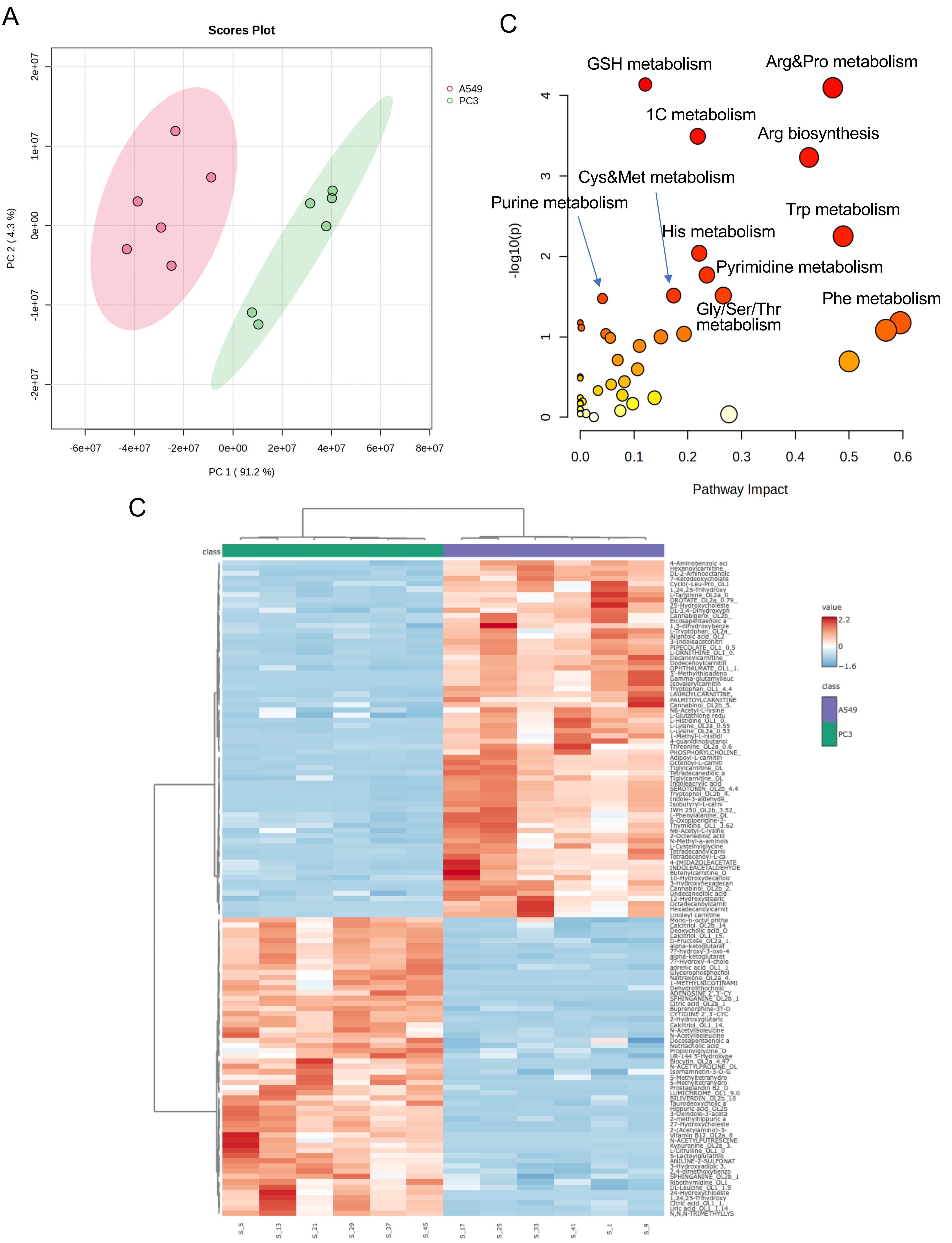

Figure S3. A549 and PC3 cells have very different metabotypes. (A) PCA for the comparison of untargeted metabolomics between A549 and PC3 cells based on 721 most confident metabolites. (B) Heat map for 125 top metabolites different between A549 and PC3 cells. (C) Pathways differentiating A549 and PC3 cells. The analysis was performed using MetaboAnalyst 6.0. For each cell line, six biological replicates were analyzed.

Supplementary Figure S4

A. A549

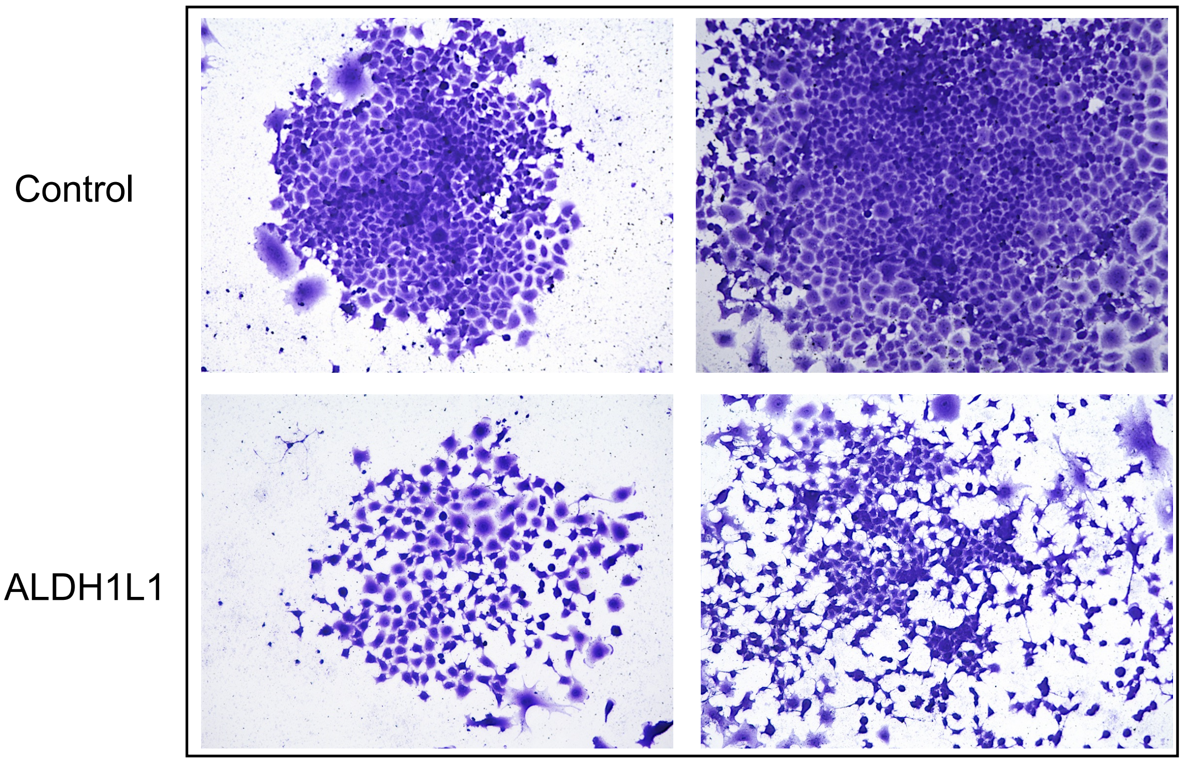

B. PC3

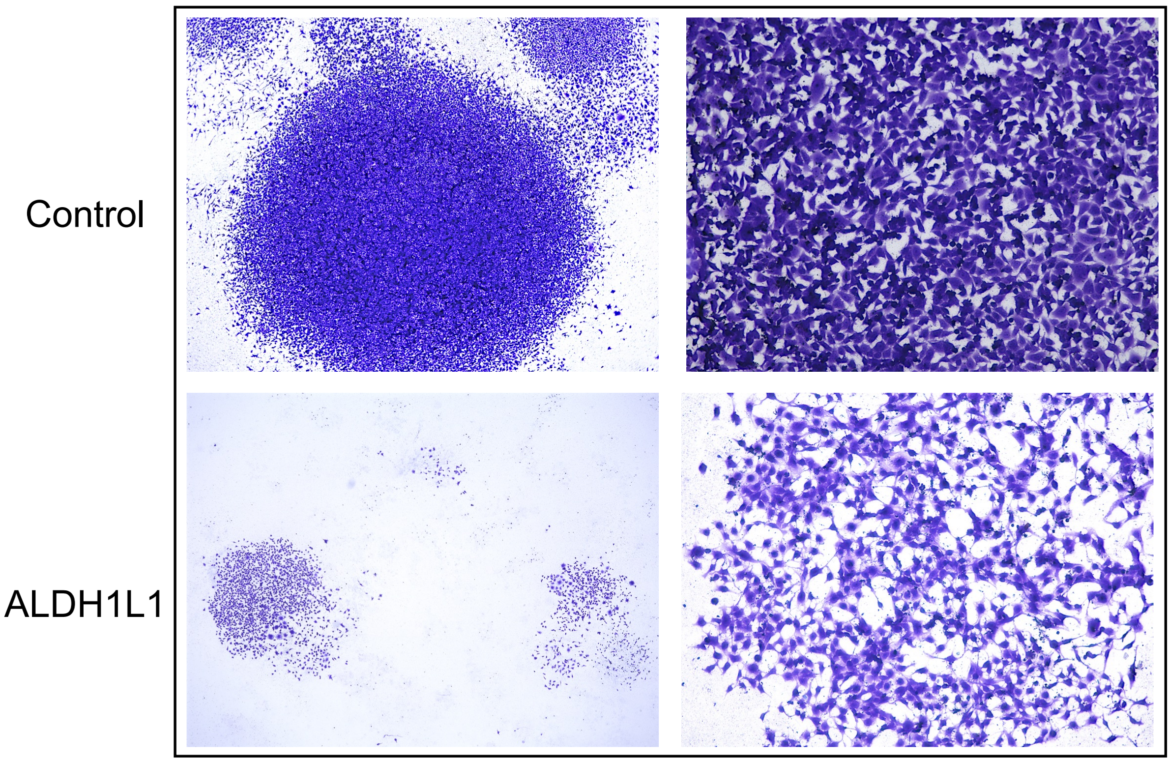

Figure S4. Representative images of colongenic assay of control and ALDH1L1 expressing, (A) A549 and (B) PC3 cells. All images were taken using Keyence BZ-X700 microscope.

Supplementary Figure S5

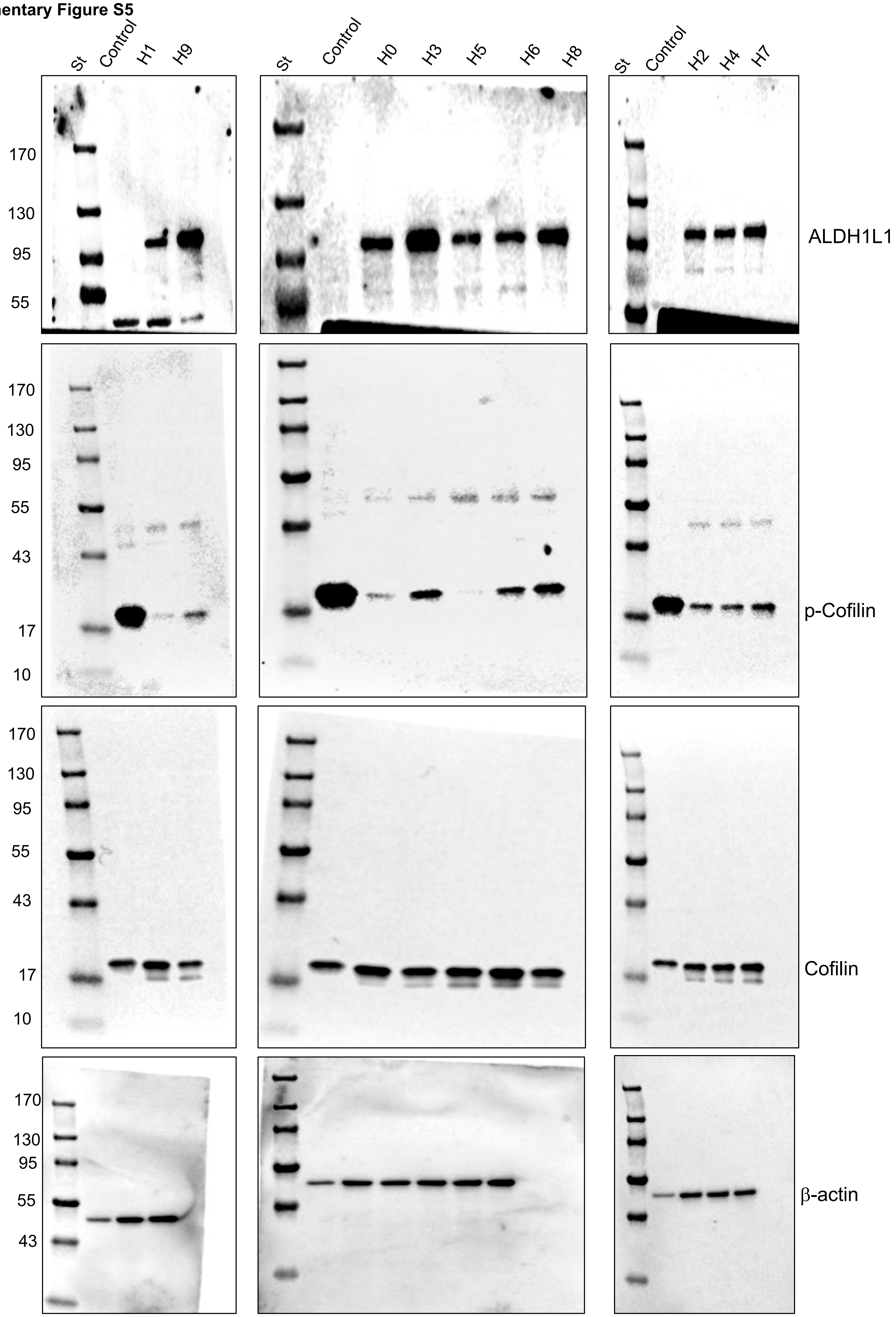

Figure S5. Original immunoblot images corresponding to Figure 8B. St, Standard protein ladder.

Supplementary Figure S6

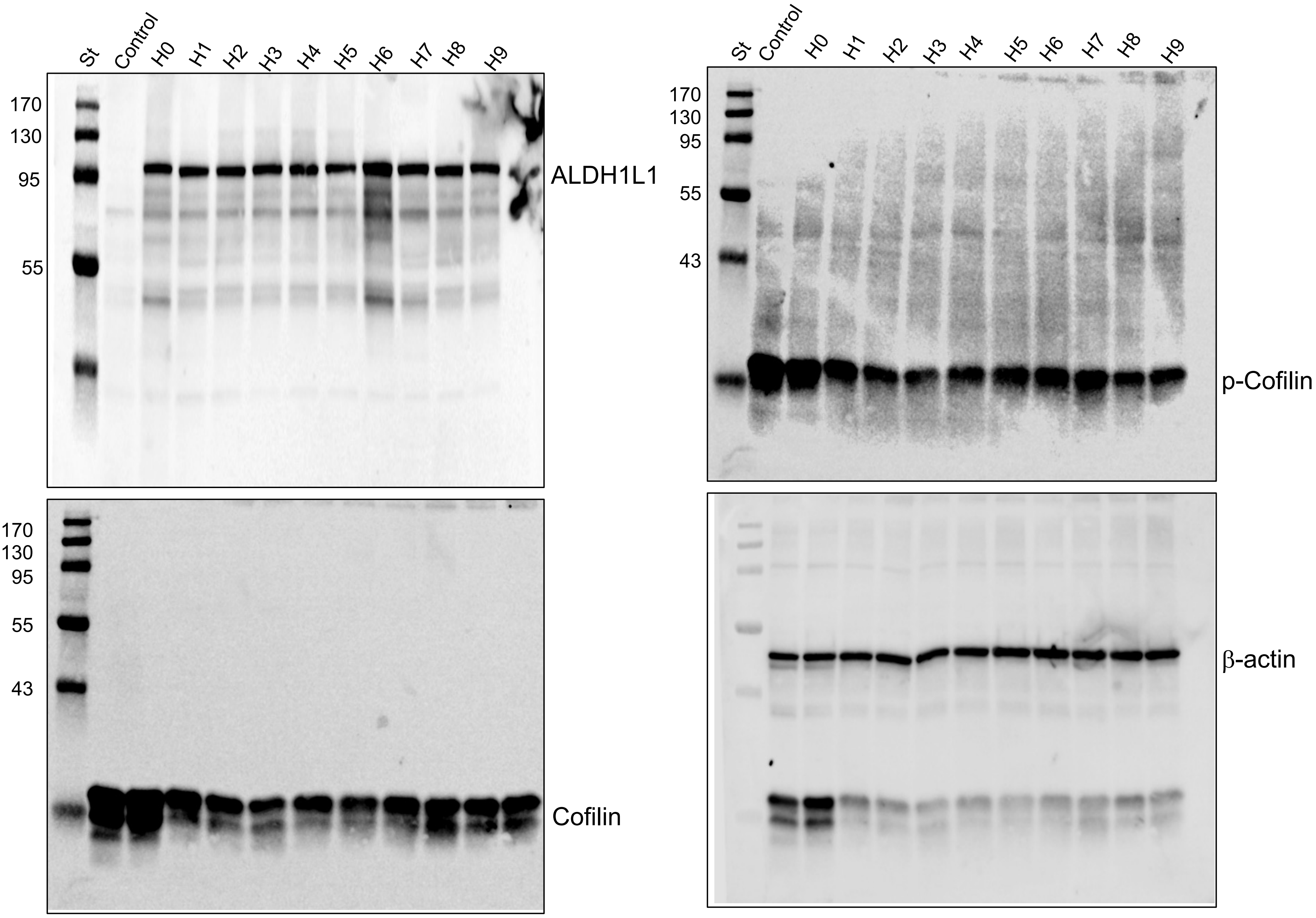

Figure S6. Original immunoblot images corresponding to Figure 9B. St, Standard protein ladder.
