# Supplementary material for: Genetic Variations in the Folate Regulatory Enzyme ALDH1L1: Impact on Cell Metabolism, Proliferation, and Motility
Source: Cells. 2026 Nov 6;15(21):2026. doi: 10.3390/cells15212026 (PMC13761728; doi:10.3390/cells15212026)
Supplement: Supplementary file 1 [file cells-15-02026-s001.zip › Sublementary Table S3_0911626 2.pdf]

Table S3. Primer sequences used for DNA mutagenesis for all ALDH1L1 SNP variants.

ALDH1L1 Mutant	Primer sequence	Annealing temperature (°C)
rs1127717 (D793G)	F: 5'-GCCAACTGTTTTCACAGGCGTGGAAGACCAC-3'<br>R: 5'-GTGGTCTTCCACGCCTGTGAAAACAGTTGGC-3'	70.8
rs3796191 (L254P)	F: 5'-CACTTCAGGCCCGGTGCCCGAGG-3'<br>R: 5'-CCTCGGGCACCGGGCCTGAAGTG-3'	71.3
rs2886059 (V330F)	F: 5'-GTGCGGAGTTTTTGGCAGCGGATC-3'<br>R: 5'-GATCCGCTGCCAAAAACTCCGCAC-3'	66.1
rs4646750 (I812V)	F: 5'-GCCTGTCATGATCGTCTCTCGGTTTGCTG-3'<br>R: 5'-CAGCAAACCGAGAGACGATCATGACAGGC -3'	69.5
rs9282691 (E429A)	F: 5'-CATTGGGGGGGCGTTCGTGGACG-3'<br>R: 5'-CGTCCACGAACGCCCCCCCAATG-3'	69.6
rs2276724 (S481G)	F: 5'-GGGGGAAGATCGGTGCGCGGGAC-3'<br>R: 5'-GTCCCGCGCACCGATCTTCCCCC-3'	73.9
