# Supplementary material for: Genetic Variations in the Folate Regulatory Enzyme ALDH1L1: Impact on Cell Metabolism, Proliferation, and Motility
Source: Cells. 2026 Nov 6;15(21):2026. doi: 10.3390/cells15212026 (PMC13761728; doi:10.3390/cells15212026)
Supplement: Supplementary file 1 [file cells-15-02026-s001.zip › Supplementary Table S1 and S2.pdf]

Table S1: ALDH1L1 Genetic Variants Identified Across the NCI-60 Cancer Cell Line Panel Using CellMiner database.

Name	Organ of origin	ALDH1L1 SNP
BT-549	Breast	rs1127717
HS 578T	Breast	rs2886059; rs2276724
MCF-7	Breast	rs3796191
MDA-MB-231	Breast	none
T-47D	Breast	rs1127717
SF268	CNS	none
SF295	CNS	rs4646750; rs2886059
SF539	CNS	none
SNB-19	CNS	none
SNB-75	CNS	none
U-251	CNS	rs2886059; rs2276724; rs4646750
Colo205	Colon	none
HCC 2998	Colon	rs4646750; rs2886059; rs1127717; rs2276724; rs3796191
HCT-116	Colon	rs1127717
HCT-15	Colon	none
HT-29	Colon	none
KM12	Colon	rs2886059; rs2276724
SW620	Colon	none
786-O	Kidney	none
A498	Kidney	rs2276724
ACHN	Kidney	none
CAKI	Kidney	rs2886059; rs1127717; rs4646750
RXF 393	Kidney	rs2276724; rs2886059
SN12C	Kidney	rs2276724; rs4646750
TK-10	Kidney	none
UO-31	Kidney	rs2276724; rs2886059
CCRF-CEM	Leukemia	rs2276724; rs2886059
HL-60	Leukemia	rs1127717
K-562	Leukemia	rs2886059; rs1127717; rs2276724
MOLT-4	Leukemia	rs1127717; rs3796191
RPMI-8226	Leukemia	rs2886059; rs1127717; rs2276724; rs9282691
SR	Leukemia	none
A549	Lung	rs1127717
EKVX	Lung	rs1127717
HOP-62	Lung	rs1127717; rs4646750
HOP-92	Lung	rs4646750; rs2886059; rs2276724
NCI-H226	Lung	none
NCI-H23	Lung	rs3796191
NCI-H460	Lung	none
NCI-H522	Lung	rs2886059; rs1127717; rs2276724
LOX IMVI	Skin	rs2886059; rs1127717; rs4646750; rs2276724
M14	Skin	rs1127717
MALME-3M	Skin	none
MDA-MB-435	Skin	rs1127717
SK-MEL-2	Skin	none
SK-MEL-28	Skin	rs1127717

SK-MEL-5	Skin	rs3796191
UACC-257	Skin	none
UACC-62	Skin	none
IGROV-1	Ovary	rs1127717
OVCAR-3	Ovary	rs1127717
OVCAR-4	Ovary	rs2886059; rs1127717; rs2276724
OVCAR-5	Ovary	rs2886059; rs1127717; rs2276724
OVCAR-8	Ovary	none
SK-OV-3	Ovary	rs1127717
NCI-ADR-RES	Ovary	none
DU145	Prostate	none
PC-3	Prostate	rs1127717

Table S2: Genetic variants (SNPs) in ALDH1L1 across tested cancer cell lines.

**Name**	**Organ of origin**	**ALDH1L1 SNP**
H596	Lung	None
H1975	Lung	rs2886059; rs2276724
H1299	Lung	None
H2110	Lung	rs2886059, rs9282691
H358	Lung	rs2886059; rs2276724, rs1127717; rs4646750
SK-MES	Lung	None
SK-BR-3	Breast	rs2886059; rs2276724
MDA-MB-468	Breast	None
RT-4	Urinary tract	None

Genomic DNA from all cell lines was extracted and analyzed using pre-designed TaqMan™ SNP Genotyping Assays. Genotyping was performed by allelic discrimination on a QuantStudio™ 12K Flex Real-Time PCR System using VIC- and FAM-labeled allele-specific probes. SNPs with a call rate <95% or ambiguous genotype clustering were excluded from further analysis.
