# Supplementary material for: Genetic Variations in the Folate Regulatory Enzyme ALDH1L1: Impact on Cell Metabolism, Proliferation, and Motility
Source: Cells. 2026 Nov 6;15(21):2026. doi: 10.3390/cells15212026 (PMC13761728; doi:10.3390/cells15212026)
Supplement: Supplementary file 1 [file cells-15-02026-s001.zip › Supplementary Table S4.pdf]

Supplementary Table S4. Statistical parameters for OPLS-DA models.

Figure	R2X	R2Y	Q2
3A	0.699	0.974	0.636
3E	0.653	0.942	0.598
5B	0.787	0.831	0.482
